# LncRNA ontology: inferring lncRNA functions based on chromatin states and expression patterns

**DOI:** 10.18632/oncotarget.5794

**Published:** 2015-09-22

**Authors:** Yongsheng Li, Hong Chen, Tao Pan, Chunjie Jiang, Zheng Zhao, Zishan Wang, Jinwen Zhang, Juan Xu, Xia Li

**Affiliations:** ^1^ College of Bioinformatics Science and Technology and Bio-Pharmaceutical Key Laboratory of Heilongjiang Province, Harbin Medical University, Nangang, Harbin, Heilongjiang, China

**Keywords:** long non-coding RNA, chromatin pattern, lncRNA ontology, lncRNA functions, integrated model

## Abstract

Accumulating evidences suggest that long non-coding RNAs (lncRNAs) perform important functions. Genome-wide chromatin-states area rich source of information about cellular state, yielding insights beyond what is typically obtained by transcriptome profiling. We propose an integrative method for genome-wide functional predictions of lncRNAs by combining chromatin states data with gene expression patterns. We first validated the method using protein-coding genes with known function annotations. Our validation results indicated that our integrative method performs better than co-expression analysis, and is accurate across different conditions. Next, by applying the integrative model genome-wide, we predicted the probable functions for more than 97% of human lncRNAs. The putative functions inferred by our method match with previously annotated by the targets of lncRNAs. Moreover, the linkage from the cellular processes influenced by cancer-associated lncRNAs to the cancer hallmarks provided a “lncRNA point-of-view” on tumor biology. Our approach provides a functional annotation of the lncRNAs, which we developed into a web-based application, LncRNA Ontology, to provide visualization, analysis, and downloading of lncRNA putative functions.

## INTRODUCTION

Recent advances in tiling arrays and RNA deep sequencing (RNA-seq), have revealed that between and within protein-coding genes there lie sequences for many thousands of long non-coding RNAs (lncRNAs) greater than 200 nucleotides (nt) in length [1]. LncRNAs affect many biological processes [2–4], including regulation of gene expression, genomic imprinting, nuclear organization, and compartmentalization. However, we have limited knowledge of how lncRNA functions [5], even for the earliest discovered lncRNAs, such as mammalian H19 [6–8], Xist [9] or HOTAIR [10].

Functional characterization of lncRNAs is a challenging task. Poor sequence conservation and tissue-specific expression make it difficult to accurately predict from the level or extent of its expression, or its sequence composition [11, 12]. In addition, there is lack of molecular interaction data, further hampering functional annotation of lncRNAs [13–15]. Genetic loss-of-function strategies can be used to study the function of lncRNAs *in vivo*, however, these are time-consuming and expensive [16]. Recently, several approaches have been proposed to predict lncRNA function, but only a small portion of lncRNAs have been functionally characterized. Cabili *et al.* has defined a reference catalog of > 8,000 human lncRNAs and functionally characterized these lncRNAs through co-expression between protein-coding genes and lncRNAs [17]. Similarly, Liao *et al.* has constructed a coding-noncoding co-expression network based on gene expression data and predicted the probable functions for lncRNAs in that network [18]. Recently, Guo *et al.* tried to apply a global network-based strategy to tackle this issue [19]. They developed a bi-colored network based global function predictor (‘lnc-GFP’) to predict probable functions for lncRNAs on a large scale by integrating gene expression data and protein interaction data. Although all of these studies have enhanced our knowledge about lncRNAs, only gene expression data and local genomic information were used in their methods [19].

Considering the key roles that lncRNAs likely play, their transcription must be tightly regulated. Similar to protein-coding genes, most lncRNAs are transcribed by RNA polymerase II and have typical epigenetic hallmarks, including DNA methylation and acetylation and/or methylation of histone residues [20]. Epigenetic-based mechanisms including DNA methylation and acetylation and/or methylation of histone residues play a critical role in gene and lncRNA expression [21, 22]. The histone modification state of genomic regions is hypothesized to reflect the regulatory activity of the underlying genomic sequence, so investigations of these features may advance our understanding of lncRNAs. Previous studies have demonstrated that chromatin marks correlate with gene expression [23, 24]. Histone modifications can either activate or repress gene transcription, and occur combinatorially to form a ‘histone code’ that is read by other proteins to give rise to various downstream events [25]. Interestingly, Wamstad *et al.* found that, despite similar expression patterns, groups of functionally related genes can be distinguished at the chromatin level [26]. Many studies have identified other noncoding regulators, such as enhancers, based on the chromatin combinations [27–29]. These results suggest that genomic annotation of these chromatin states can extend the functional interpretation of noncoding part of the human genome. Based on these observations, a number of studies have used chromatin patterns to identify lncRNAs. Guttman *et al.* systematically discovered a large number of lncRNAs by exploring chromatin structure and developed an approach to predict putative functions [30]. By the same method, Khalil *et al.* also identified approximately 3,300 lincRNAs in six human cell types and further examined the associations between these lincRNAs and polycomb repressive complex 2 (PRC2) [31]. In addition, Lv *et al.* demonstrated that the accuracy of lncRNA predictions can be greatly improved when incorporating chromatin modifications data [32]. Moreover, Ounzain *et al.* reasoned that lncRNAs that share specific chromatin patterns as those described for coding genes are likely to be involved in comparable biological processes. Based on this hypothesis, they successfully inferred heart-specific functions for the novel lncRNAs identified in their study [33].

**Figure 1:**
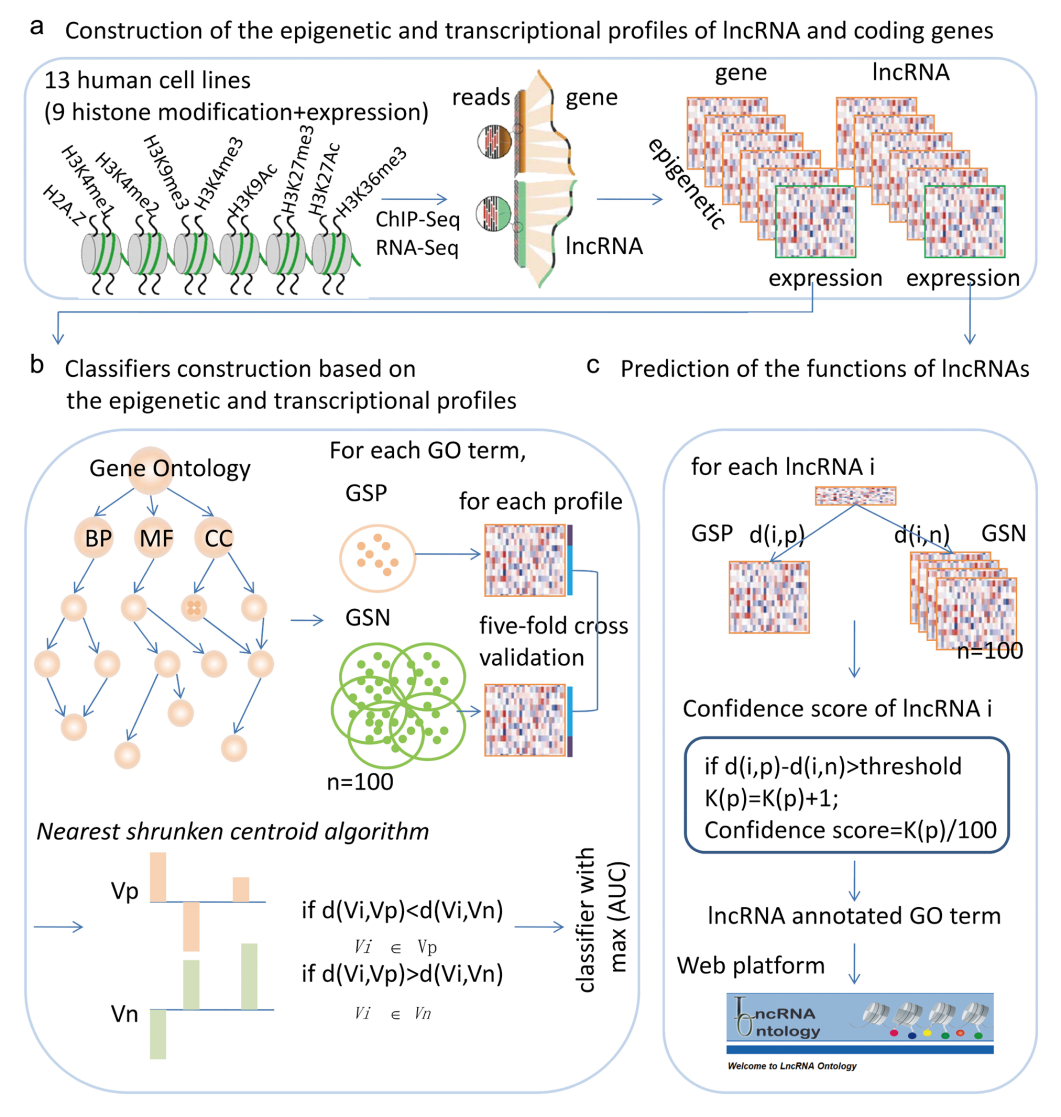
Workflow for predicting the functions of lncRNAs based on chromatin and expression patterns **a.**, chromatin and expression profiles for lncRNAs and protein coding genes were constructed based on ChIP-Seq and RNA-Seq datasets. **b.**, for each GO term and each chromatin and expression profile, a nearest shrunken centroid algorithm based classifier was constructed and the power of the classifiers was evaluated by AUC. The classifier with the maximum AUC was selected as the final classifier. **c.**, predicting the functions of lncRNAs based on the chromatin and expression patterns.

Motivated by these studies, here, we propose an integrative framework to predict the lncRNA functions based on both chromatin states and exprssion patterns (Figure 1). Taking advantage of the datasets from the ENCODE project, we compiled genome-wide chromatin and expression profiles for lncRNAs and coding genes. We then derived a novel unbiased integrative model to functionally annotate lncRNAs. The proposed method was validated on protein-coding genes with known functional annotations by five-fold cross-validations. Applying the trained integrative model, we predicted the probable functions for more than 97% of human lncRNAs. We also linked cancer-associated lncRNAs to cellular processes that are hallmarks of cancer, providing a “lncRNA point-of-view” on tumor biology. Our attempt to compile massive RNA-Seq and ChIP-Seq data will facilitate future functional investigation of lncRNAs and serves as an important resource (LncRNA Ontology), for further biological research.

## RESULTS

### Integration of transcriptional and chromatin features effectively predicts gene functions

**Figure 2:**
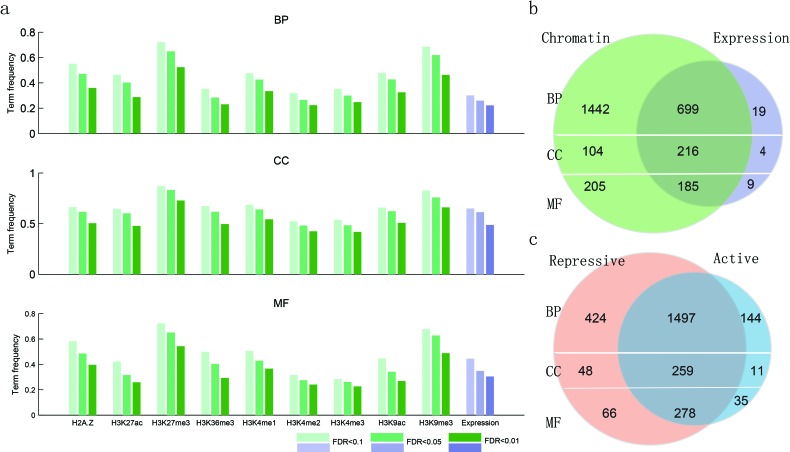
Functionally similar genes have epigenetic and transcriptional similarities **a.**, the proportion of GO terms with epigenetic and transcriptional similarities. **b.**, venn diagram showing the number of GO terms with chromatin and expression similarities. **c.**, venn diagram showing the number of GO terms with chromatin similarities, with the chromatin states divided into active and repressive based on their effects on gene expression.

To systematically study the relationship between biological function and chromatin state, we collected 117 ChIP-seq profiles of histone modifications (seven activating signals and two repressive ones) along with the corresponding transcriptome data assayed by RNA-seq across thirteen human cell lines. These data were downloaded from the ENCODE project (Table S1). We hypothesized that groups of functionally related genes (as determined by gene ontology [GO]) would have similar histone modifications and expression levels, which could be used to distinguish them. For genes within each GO term from the database, we calculated their similarity at the level of expression and modification. We found that genes annotated in the same term of the biological process (BP) ontology show high co-expression at the FDR < 0.01; however, just 20% BP terms were satisfied. This is similar to what has been found in *S. cerevisiae* and *C. elegans* [34], indicating that co-expression alone generally provides a relatively narrow range for functional prediction. In contrast, compared to co-expression, we found a much wider similarity at the level of epigenetic modifications under different FDR threshold values (Figure 2a). Moreover, the vast majority (97.17%) of GO terms with high co-expression also show high chromatin similarity (Figure 2b). The same tendency was also found for two additional ontology branches. In addition, there are many GO terms that were only similar at the level of chromatin modifications, suggesting that expression was not the best predictor for these terms. Interestingly, complementary effects were revealed for active and repressive chromatin modifications, with many functional groups exhibiting high similarity in both kinds of modifications (Figure 2c).

**Figure 3:**
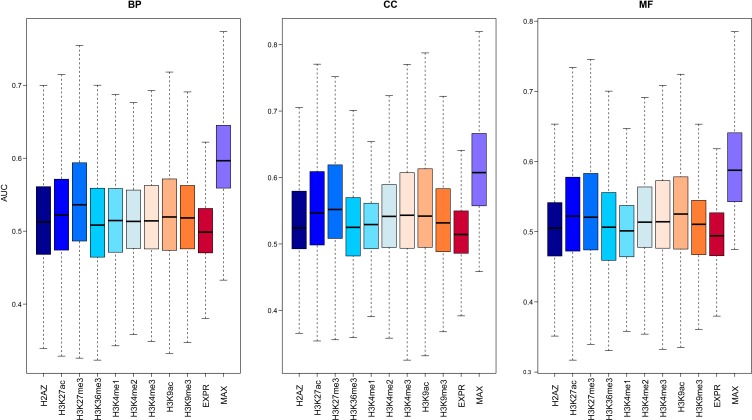
The predictive power of the model using signals from different chromatin or expression features For each GO term, 100 GSNs with the same number of genes as GSPs were randomly selected from the remaining genes, and then cross-validation was used to compute the AUC. The average AUCs for 100 times of all GO terms are shown in the boxplot. The left panel is for biological process (BP) categories, middle panel for cell component (CC) categories and the right panel for molecular function (MF) categories.

Given that both gene expression and epigenetic regulation can act as predictors for function of protein-coding genes, we wanted to establish an integrative method using both expression and modifications to predict lncRNA functions genome-wide. First, we trained this model on protein-coding genes with known functional information (Materials and Methods). We compared the predictive power of 9 chromatin features and gene expression using the index of average Area Under the Curve (AUC). As shown in Figure 3, all the chromatin and expression features were much better predictors than random ordering, which would give an expected AUC of 0.5. The median AUCs of the models learned from the chromatin features were all higher than those for expression, and there was no difference in performance between active and repressive chromatin modifications. The relative predictive power of different chromatin features was heterogeneous across different biological processes. Some GO terms with higher AUCs learned from chromatin features while others learned from expression. These results suggested that the integration of these two predictors was best; therefore, we used an integrative model to predict gene functions for each specific GO term. The median AUC value of all terms was ∼0.6, comparable to the performance of the top-five performing models in the CAFA experiment [35], validating our integrative model as an effective predictor of gene function.

### Integration of transcriptional and epigenetic features predicts the functions of lncRNAs

**Figure 4:**
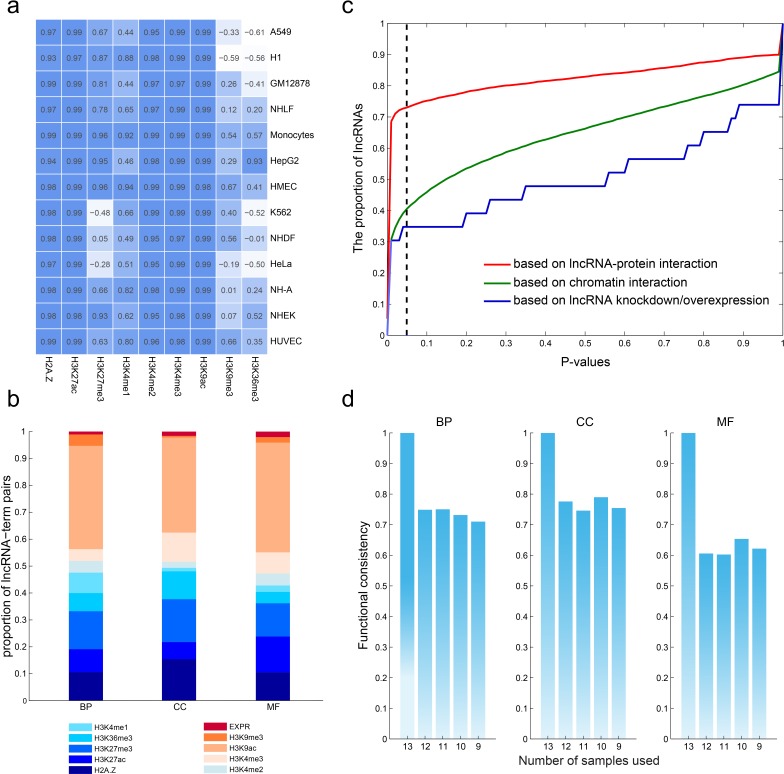
Robustness of the predicted functions of lncRNAs **a.**, lncRNAs share common chromatin patterns with protein coding genes. The matrix shows the correlation of chromatin modification around the TSSs (+2kb) of lncRNAs and protein coding genes in each cell type. **b.**, the contribution of each chromatin and expression feature to the predicted lncRNA-GO term associations. **c.**, the consistency of lncRNA-GO term associations using different number of cell types. **d.**, predicted lncRNA functions show high consistency with commonly used methods.

The promoters of genes and lncRNAs were divided into 20bp bins and read density was counted. We observed that, as shown previously, several histone marks show a similar distribution pattern around the transcriptional start site (TSS) of both lncRNAs and mRNAs irrespective of cell types. This was particularly true for the activating signals, H3K4me3 and H3K4me1, as well as the repressive histone mark H3K27me3 (Figure 4a and Figure S1-S13). These findings suggest that the epigenetic regulation of lncRNAs is similar to that of protein coding genes.

Based on our prior hypothesis about predicting function of protein-coding genes, we predicted lncRNA functions using these same integrative modeling. We used the nearest shrunken centroid algorithm to assign lncRNAs to each GO term with a confidence score ranging from 0 to 1 (Materials and Methods). We obtained 5,404,928 lncRNA-GO associations among 17,998 lncRNAs and 1,256 GO terms with a confidence score of 1.0, and we assigned functions for 97.79% of all lncRNAs for BP, 97.77% of all lncRNAs for cellular components (CC), and 97.77% of all lncRNAs for molecular function (MF). LncRNAs were predicted to be involved in diverse biological processes including organ/tissue development (e.g. neuron, eye and muscle development), cellular transport, and metabolism. We next analyzed how much each predictive feature contributed to lncRNA functional associations. As shown in Figure 4b, H3K9ac contributed the highest number of associations, while the lncRNA expression contributed the least. H2A.Z, H3K27ac, H3K36me3 and H3K4me1/3 are comparable in the number of the predicted results. The above analyses suggest that the functions of lncRNAs can be at least partially deduced from chromatin features.

### An integrative model provides a robust method of predicting lncRNA functions

While only a small number of lncRNAs have been functionally characterized, it is believed that lncRNAs interact with DNA, RNA, and proteins [34], acting as regulators in chromatin organization, transcription and post-transcriptional modulation. In order to validate our predictions of lncRNA function, we tested our findings against known lncRNA-protein interactions, lncRNA-chromatin interactions, and lncRNA knockdown/overexpression datasets. First, we collected the lncRNA-protein interactions identified by CLIP-Seq datasets and predicted the functions of lncRNAs by annotation of the interacting genes. Based on these interactions, 4,505 lncRNAs regulated at least one function, whereas our model predicted the same functions for 3,291 (73.05%) of these lncRNAs (Figure 4c, *P* < 0.05). The 3D structure of the genome plays a critical role in regulating gene expression [36]; thus, we compiled the chromatin interaction datasets from the 4Dgenome database, involving 616,476 interactions among 12,434 lncRNAs and 17,451 genes. The predicted functions of 40.65% of lncRNAs overlapped with those predictions from our integrative model. Finally, we utilized data from lncRNA knockdown/overexpression experiments collected from the LncRNA2Target database [16], where the differentially expressed genes are considered as the target genes of the lncRNAs. Based on these datasets, we found the functions were matched with our predictions for 8/23 (34.78%) of lncRNAs.

As these methods predicted the functions of lncRNAs from different viewpoints, we analyzed these results and found that most of the unique lncRNA-GO term pairs were predicted by our method (Figure S14). These pairs provide candidates for further experimental validation. In addition, we observed that a majority of pairs were predicted by H3K9ac histone marks. Recently, H3K9ac was shown to have a high power to predict the expression of genes [24], suggesting that H3K9ac plays a key role in regulating gene expression. We also observed that about 91 pairs of lncRNA-GO terms were included in the lncRNAdb v2.0 [37], a comprehensive, manually curated reference database of lncRNAs that have been described independently in the scientific literature.

Taken together, our results indicate the robustness of our model at predicting lncRNA functions. To further assess our model based on available datasets, we gradually reduced the number of cell lines from 13 to 9 to perform the above prediction pipeline. When sample size decreased, the median AUC pattern never changed (Figure S15-Figure S18). In addition, analysis of the lncRNA-function pairs at varying sample numbers revealed up to 80% functional consistency across all three ontologies at a confidence score of 1.0 (Figure 4d). These analyses indicate that sample size may not be a significant part of our algorithm's final performance.

### Prediction of functions for cancer-associated lncRNAs

**Figure 5:**
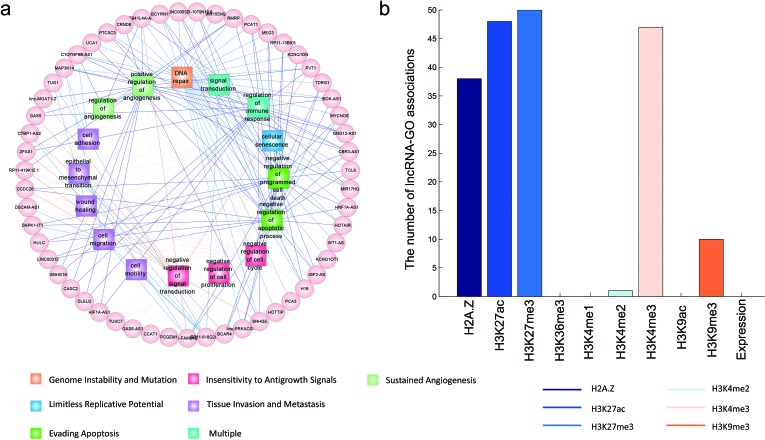
The predicted functions of cancer-associated lncRNAs **a.**, the lncRNA-GO functional associations are shown as a network. Only GO terms associated with cancer hallmarks are shown, and the GO terms with the same hallmarks are shown in the same color. The color of edges indicatethe type of histone modification used to predict the functional associations. **b.**, The number of lncRNA-GO term associations predicted by different chromatin and expression features.

LncRNAs are dysregulated in several human cancers and involved in a broad spectrum of functions [38, 39]. Although the biology of cancer is extremely complex, there are a few cancer hallmarks that enable tumor growth and metastasis dissemination [40, 41]. Here, we linked the cellular processes influenced by lncRNAs to the hallmarks of cancer, providing a “lncRNA point-of-view” on tumor biology. We used a list of GO terms previously defined as related to cancer hallmarks [42] and obtained 109 experimentally-validated cancer lncRNAs, including disease lncRNAs in the LncRNADisease database [43]. Of these lncRNAs, we found that 55 were associated with at least one cancer-associated GO term. And these lncRNAs are totally functionally annotated with 19 cancer hallmark GO terms. Figure 5a shows the 194 pairs of lncRNA-GO associations. With analysis of chromatin and expression patterns, we found that the 194 pairs of lncRNA-GO associations were predicted based on chromatin similarity (Figure 5b). Some functional links were consistent with the literature. For example, HOTAIR is a well-known lncRNA whose dysregulation correlates with poor prognosis and malignant progression in many forms of cancer [44, 45]. Knockdown of HOTAIR results in the induction of cell cycle arrest and apoptosis [46]. Based on chromatin patterns, we observed that HOTAIR was associated with DNA repair and the regulation of apoptosis. Another example is the lncRNA, MEG3, which is highly expressed in non-neoplastic tissues, but lowly expressed in cancer tissues. Ectopic expression of MEG3 inhibits the proliferation of cervical carcinoma cells through the induction of cell cycle arrest and apoptosis [47]. We found that, based on H3K4me3 and H3K27ac patterns, MEG3 is involved in the ‘negative regulation of cell cycle’ and ‘negative regulation of apoptotic process’. These results identify an important role of MEG3 in the molecular etiology of cancer and implicate it as a potential target for cancer therapy.

### LncRNA Ontology: a database of lncRNA functions

Based on our data, we have developed a free, web accessible database, LncRNA Ontology (http://www.bio-bigdata.com/lncrnaontology/), which makes the functions, chromatin, and expression patterns viewable to users across cell types. In addition, we also annotated the lncRNA-GO pairs predicted based on chromatin interaction, lncRNA-gene interaction, and lncRNA over-expression or knockdown data, as well as those lncRNA-GO pairs reported in the literature. LncRNA Ontology can support rapid searches by individual lncRNA or by a specific GO term, and allows for data downloads. It currently provides the functions predicted for each lncRNA in our study: 5,404,928 lncRNA-GO associations among 17,998 lncRNAs and 1,256 GO terms.

## DISCUSSION

Although mammalian cells produce many thousands of lncRNAs, the functional significance of these transcripts has been controversial. Co-expression networks of genes and lncRNAs, in which a node represents a gene or lncRNA and an edge represents an expressional correlation, have been used to identify cellular modules and predict the functions of unknown lncRNAs [48–50]. In addition, genome-wide chromatin-state maps provide another rich information source about cellular state, yielding insights beyond what is typically obtained by transcriptome profiling. Genes with similar expression patterns could share a common chromatin pattern or may be represented by multiple different chromatin patterns [23]. However, chromatin information had been ignored when predicting the functions of lncRNAs.

In this study, we showed that chromatin modifications and gene expression are strongly correlated with gene function. Although most of genes within a GO term show both highly similar chromatin marks and expression patterns, some GO terms only show chromatin modification similarity. Previous studies have proposed that some histone modifications are the memory of past transcriptional events resulting from previous active transcription [51, 52]. Other studies have shown that chromatin modification changes precede changes in gene expression [53]. For instance, a recent study in human T cells demonstrated that, for both protein-coding and non-coding RNAs, activating histone marks were already in place before the induction of expression, and these marks were maintained even after the genes were silenced [54]. Thus, these epigenetic signals may be helpful in the functional annotation of genes or lncRNAs, beyond the functions predicted based on expression patterns. Using both gene expression and chromatin data, we developed a novel integrated model to predict the functions of lncRNAs. We reasoned that lncRNAs that shared specific chromatin patterns as those coding genes with functional annotations were likely to be involved in comparable biological processes. We used ENCODE data to reveal that chromatin patterns can predict sets of functionally related genes, which implies that functionally related genes have specific modes of epigenetic regulation. Moreover, we showed that, in terms of function, information from different histone modifications is considerably more effective at predicting function than gene expression, and that integration of chromatin features and expression patterns can predict the functions of protein coding genes with high accuracy.

Although our strategy was successful at predicting functional annotations of lncRNAs, our method can be improved in several ways. First, in this study, we made our best effort to collect a number of samples with histone modifications. Although we have demonstrated that the accuracy of our model is robust regardless of the number of samples, as the data become more comprehensive, extended range of potential functions will be reliably ascribed to a given lncRNA. Secondly, while we used GO function categories to annotate the lncRNAs in the study [55], the relationships among GO terms may lead to correlated functional annotations for lncRNAs. Alternatively, the function classification for lncRNAs may be not be feasible based on current knowledge. Other functional labels, such as pathway information could be characterized for lncRNAs in the future.

Taken together, integrating the chromatin and expression patterns, we generated biologically meaningful functional annotations for lncRNAs genome-wide. Our model illustrates the power in functional prediction of lncRNAs, and this study opens up new avenues to study and functionally characterize lncRNAs. We anticipate that in the future, the integration of computational function prediction and more knockout or over-expression experiments will offer even deeper insight into the lncRNA functions.

## MATERIALS AND METHODS

### Genomic annotation of lncRNAs and protein-coding genes

The genomic annotations of lncRNAs were compiled from Gencode [11], Ensembl and the study of Cabili *et al.* [17]. If the lncRNAs from Cabili *et al.* had > 80% overlap with those from Gencode or Ensembl, we retained the lncRNA annotation from Gencode or Ensembl. The annotation of protein coding genes was retrieved from the UCSC Genome Browser (Refseq table) for the hg19 build of the human genome [56]. In total, the annotation information of 18,405 lncRNAs and 44,331 gene transcripts were obtained.

### Chromatin profiles of lncRNAs and mRNAs

We compiled the genomic distributions of nine histone modifications from the ENCODE project, including H2A.Z, H3K4me1/2/3, H3K9me3, H3K27me3, H3K27ac, H3K36me3 and H3K9ac in 13 human cell lines (Figure 1a and Table S1). To aviod the bias of datasets provided by different organizations, all ChIP-seq data used were generated at the Broad Institute. We directly downloaded the mapped files in bam format. Sequence reads from each experiment were aligned to the human reference genome (GRCh37/hg19) and count coverage within a 4kb region centered at the TSS of each lncRNA/gene transcript was calculated using BEDTools multicov, a BAM focused tool [57]. The raw read counts were divided by the total number of million mapped reads in each sample (Reads Per Million, RPM) [26]. The chromatin level of each lncRNA or gene was defined as the highest value observed across all transcripts of a gene. The lncRNAs or genes that had at least one chromatin mark in one cell type were subsequently analyzed, and the epigenetic intensity of each lncRNA/gene was log2 transformed and Z-score normalized. As a result, 16,112 lncRNAs and 22,524 genes in average were analyzed for chromatin patterns.

### Transcriptional profiles of lncRNAs and mRNAs

Gene expression of 9 human cell lines were measured by RNA-seq technology, which were also downloaded from the ENCODE project (Table S1). The raw reads were downloaded and aligned to the hg19 version of the human genome using TopHat2 with default options [58]. Cufflinks was used to generate the gene-level read counts and estimate the fragments per kilobase of exon model per million mapped reads (FPKM) [59]. Only lncRNAs/genes expressed in all cell types were considered for subsequent analysis. Expression data was log2-transformed and Z-score normalized. In total, 4,453 lncRNAs and 20,746 genes with expression were analyzed in this study.

### The functional annotation of protein coding genes

The gene2go table was downloaded from the National Center for Biotechnology Information, and we extracted human related information including three different ontologies-biological process (BP), molecular function (MF) and cellular component (CC). Considering the hierarchical nature of GO categories, we considered all the descendant nodes one level below when computing the size of a node. The topology of the ontology was downloaded from GO website. GO terms were considered only if the number of annotated genes was less than 2000 but more than 20. We retained 2,385, 339 and 436 terms for BP terms, CC terms and MF terms, respectively.

### Epigenetic and transcriptional similarities of genes within the same function terms

To compute the epigenetic and transcriptional similarity among genes of each GO term, their corresponding epigenetic profiles or expression profiles were extracted, and then we calculated the Pearson correlation coefficients for every gene pair. We took the average value of all the coefficients to represent the similarity of each GO term. In order to measure the significance of the similarity, we randomly selected the same number of genes and recalculated the similarity of the GO term. The procedure was repeated 100 times. The significance was defined as the proportion of times in which in random conditions, the similarity values were higher than the real ones.

### Classifier construction based on the chromatin states and expression patterns

#### Gold standard

For each GO term, gold standard positives (GSP) were defiend as genes annotated in the GO term or all the descendant terms. However, it was difficult to obtain the gold-standard negatives (GSN). Here, the GSN was with the same gene number as the GSP, which was randomly selected from the remaining gene sets. Both GSPs and GSNs should exist in the chromatin and expression profiles. We constructed 100 GSNs for each GO term.

#### Classification algorithm and assessment

The nearest shrunken centroid algorithm was used to construct a classifier to distinguish genes with the same functions from randomly selected gene sets, incorporating the expression profile and the 9 chromatin profiles (Figure 1b). For each GO term, a classifer was constructed based on each feature profile. The performance of each classifier was evaluated through five-fold cross-validation. We split the GSPs and GSNs at random into five approximately equal-size parts, where four folds were used to create two centroids (‘positive’ and ‘negative’) using the mean profile (chromatin or expression) of the mRNAs. And then for each gene *i* in the training set, the distance difference *Δd* to the two centroids were calculated as follow:

$$\Delta d(i)=sqrt(\sum_{k-1}^{n}{\left(p_{k}(i)-GPS_{k}\right)}^{2})-sqrt(\sum_{k-1}^{n}{\left(p_{k}(i)-GSN_{k}\right)}^{2})$$

where *p*(*i*)=(*p*_1_(*i*) *p*_2_(*i*), … *p*_*n*_(*i*)) is the chromatin or expression profile of gene *i* in *n* samples, (*GSP_1_, GSP_2_, … GSP_n_*) and (*GSN_1_, GSN_2_, … GSN_n_*) are the two centroids of the GSP and GSN, and *n* is the size of the feature profiles. Then, if the *Δd* of gene *i* is higher than the given cutoff, we proposed that the gene was predicted to annotate to this functions, otherwise the gene was not predicted to be involved in the function. This procedure was repeated five times. The quality of the classifier was evaluated by plotting the ROC curve at various cutoffs of Δ*d*. The ultimate performance for each function term was evaluated by the average AUC because of 100 constructed GSNs.

### Prediction the functions regulated by lncRNAs

As shown in Figure 1c, for each GO term, the related lncRNAs were identifed based on the classifier with the maximum AUC, which was reconstructed based on the whole GSP and GSN datasets to incorporate more information. Then the distance difference Δ*d* from the feature vector of lncRNA to the two centroids was calculated as described above. The cutoff of Δ*d* was determined by the Youden's J statistic [60]. Finally, if Δ*d* was higher than the cutoff, we proposed that the lncRNA was associated with this function, otherwise the lncRNA did not regulate the function. Corresponding to the 100 randomly selected GSNs, the classifier was carried out 100 times. Finally, the times of positive predictions were defined as the confidence score. The higher the score was, the more likely the lncRNA can regulate this function.

### Epigenetic similarity of mRNAs and lncRNAs across TSS

The genomic regions around the TSS (−2kb to 2kb) were divided into bins with 20bp, and then we counted the number of reads in each bin. For the calculation of densities over a defined window, the methods were derived from the one generally used to generate density files. And then the average read density of all genes and lncRNAs were computed for each bin. All these processes were performed by the software seqMINER [61]. And then we computed the correlation coefficient of the gene and lncRNA using the averge read density across 200 bins.

### Compared with other methods

We compared the predicted model in our study with two other commonly used methods: (1) based on the genes interacting with lncRNAs; (2) based on the genes differentially expressed after knockdown or overexpress of lncRNAs. The lncRNA-interacting genes were obtained from starBase and 4DGenome, and the differentially expressed genes were downloaded from LncRNA2Target. Then the functions of lncRNAs were predicted by the interacting genes or differentially expressed genes. A hypergeometric test was used to evaluate the function consistence of lncRNAs.

### The web development of the LncRNA Ontology

The LncRNA Ontology web interface (abbreviated to LO, http://www.bio-bigdata.com/lncrnaontology/) was developed in Java Servlet framework and deployed in tomcat 6.0.33 web server and runs under Cent OS 5.5 system. It is supported by a MySQL database of histone modification and expression data. LncRNA ontology is fully tested in Google Chrome (version 17 and later).

## SUPPLEMENTARY MATERIAL FIGURES AND TABLE




