# Supplementary material for: LncRNA ontology: inferring lncRNA functions based on chromatin states and expression patterns
Source: Oncotarget. 2015 Sep 22;6(37):39793–805. doi: 10.18632/oncotarget.5794 (PMC4741861; doi:10.18632/oncotarget.5794)
Supplement: Supplementary file 1 [file oncotarget-06-39793-s001.pdf]

LncRNA ontology: inferring lncRNA functions based on chromatin states and expression patterns

Supplementary Material

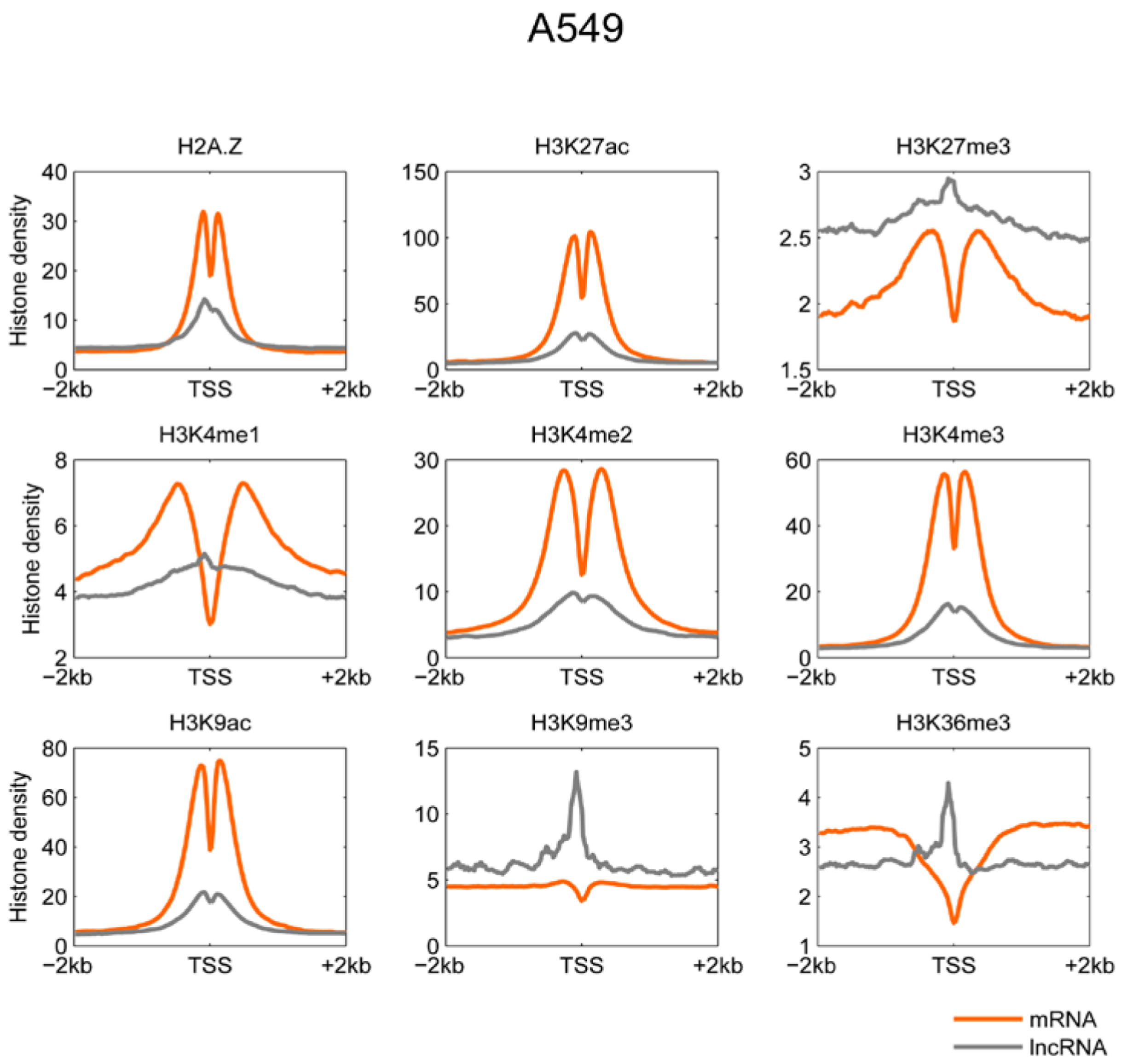

Figure S1 -The lncRNAs share common chromatin patterns with protein coding genes in A549 cell line.

The genomic regions around the TSS (-2kb to 2kb) were divided into bins with 20bp, and the number of reads in each bin was counted. Then the average read density of all genes and lncRNAs were computed for each bin. And we computed the Pearson correlation coefficient of the gene and lncRNA using the averge read density across 200 bins in figure 4A.

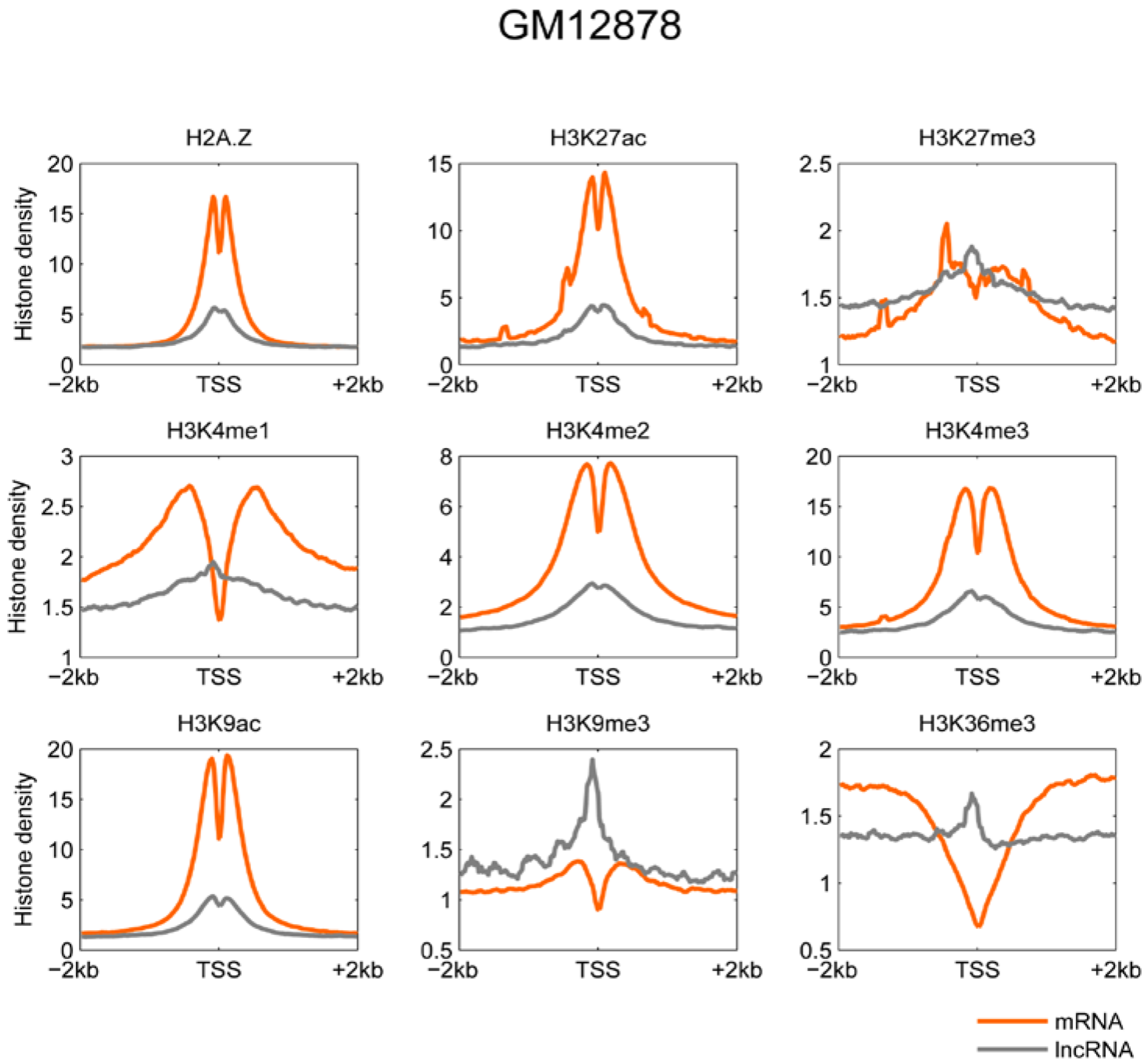

Figure S2 - The lncRNAs share common chromatin patterns with protein coding genes in GM12878 cell line.

The genomic regions around the TSS (-2kb to 2kb) were divided into bins with 20bp, and the number of reads in each bin was counted. Then the average read density of all genes and lncRNAs were computed for each bin. And we computed the Pearson correlation coefficient of the gene and lncRNA using the averge read density across 200 bins in figure 4A.

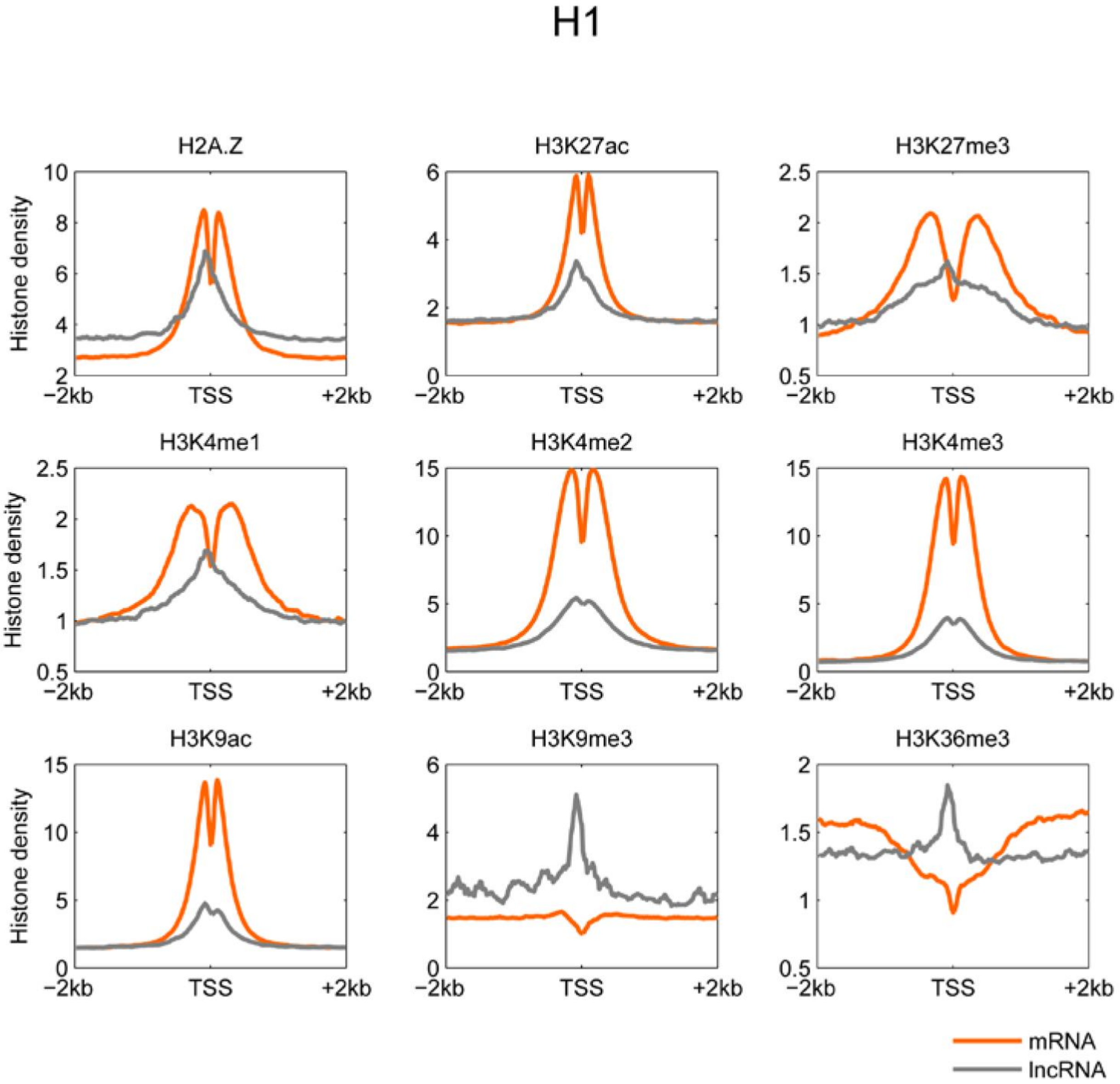

Figure S3 - The lncRNAs share common chromatin patterns with protein coding genes in H1 cell line.

The genomic regions around the TSS (-2kb to 2kb) were divided into bins with 20bp, and the number of reads in each bin was counted. Then the average read density of all genes and lncRNAs were computed for each bin. And we computed the Pearson correlation coefficient of the gene and lncRNA using the averge read density across 200 bins in figure 4A.

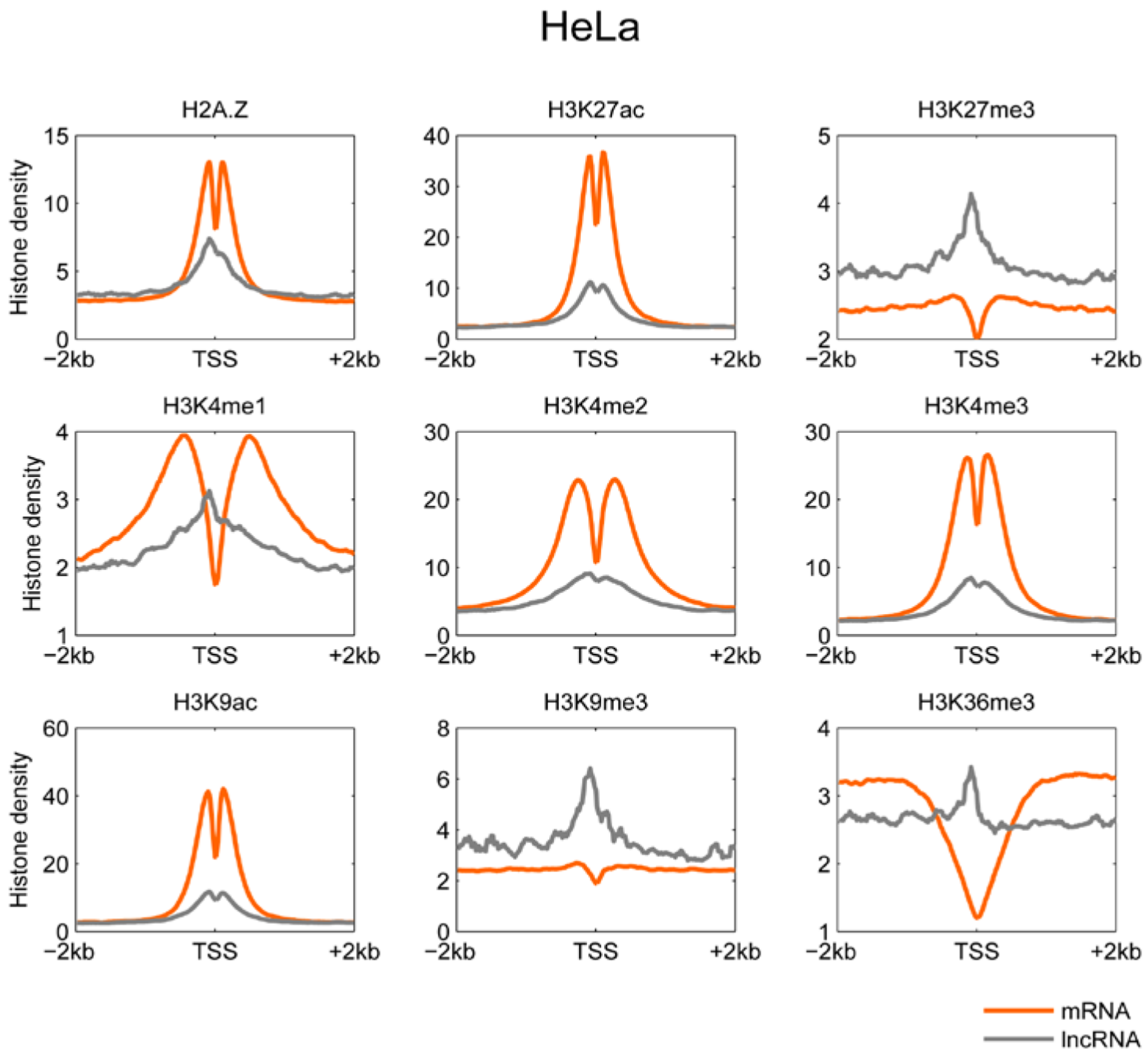

Figure S4 - The lncRNAs share common chromatin patterns with protein coding genes in HeLa cell line.

The genomic regions around the TSS (-2kb to 2kb) were divided into bins with 20bp, and the number of reads in each bin was counted. Then the average read density of all genes and lncRNAs were computed for each bin. And we computed the Pearson correlation coefficient of the gene and lncRNA using the averge read density across 200 bins in figure 4A.

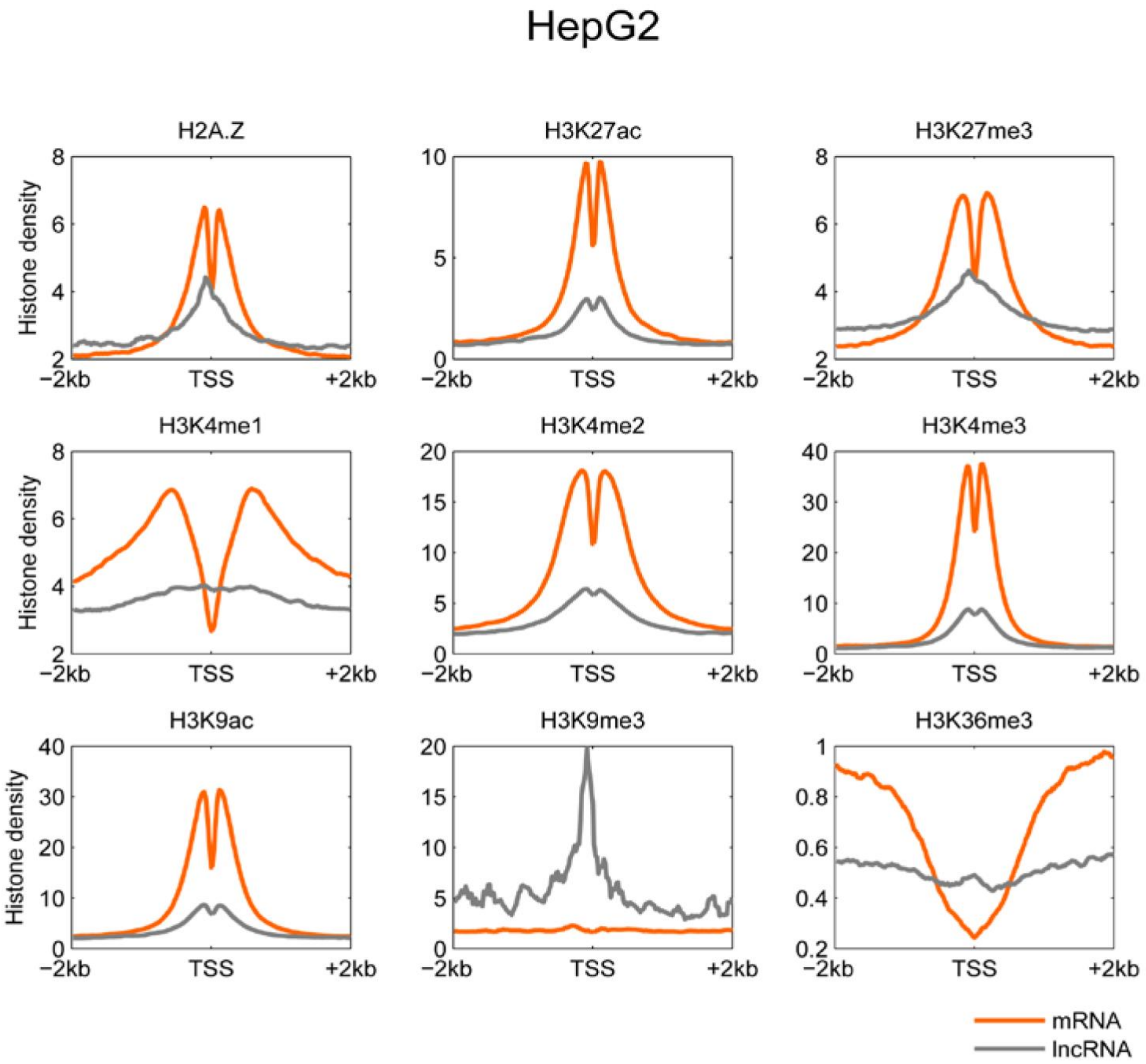

Figure S5 - The lncRNAs share common chromatin patterns with protein coding genes in HepG2 cell line.

The genomic regions around the TSS (-2kb to 2kb) were divided into bins with 20bp, and the number of reads in each bin was counted. Then the average read density of all genes and lncRNAs were computed for each bin. And we computed the Pearson correlation coefficient of the gene and lncRNA using the averge read density across 200 bins in figure 4A.

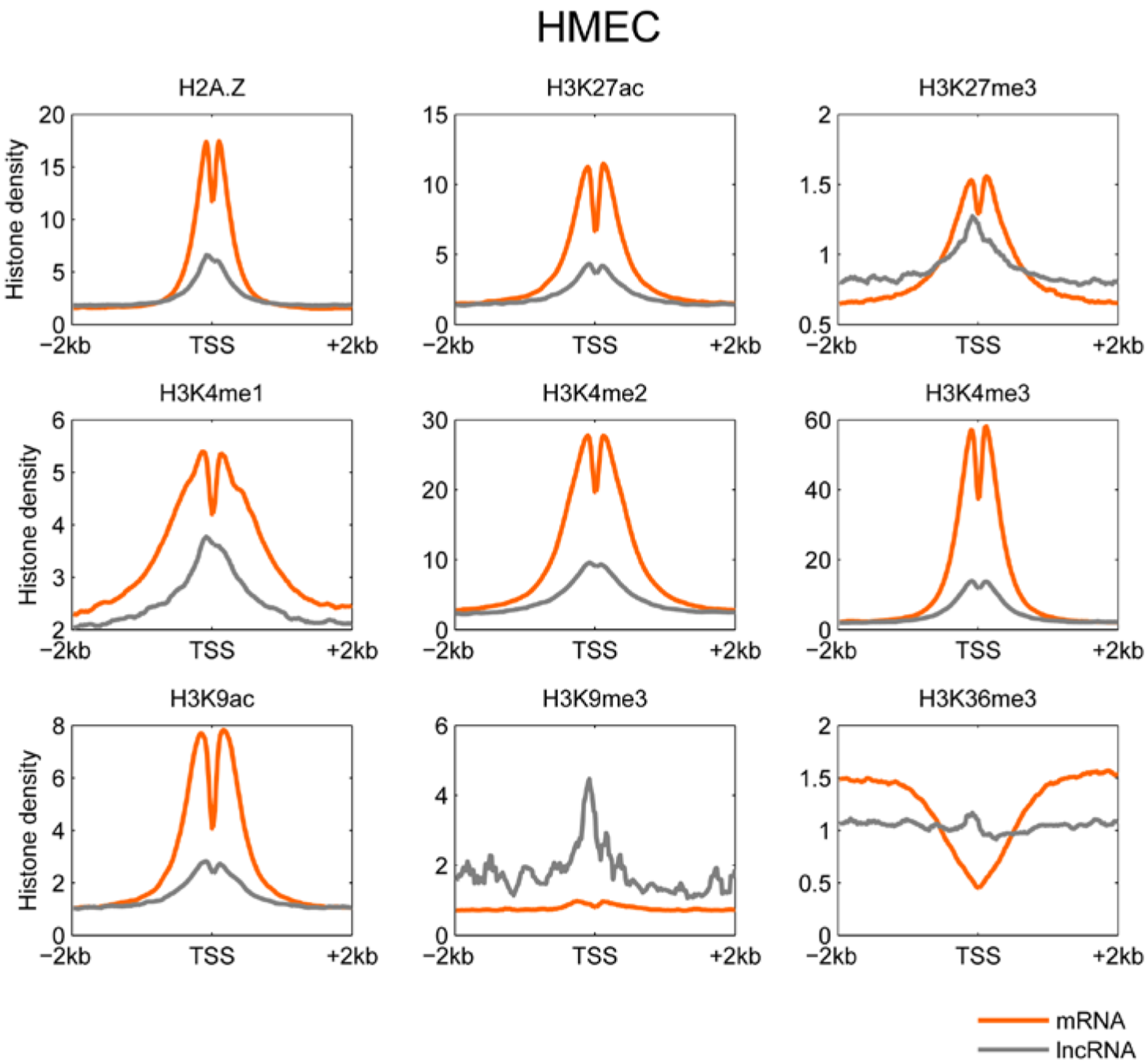

Figure S6 - The lncRNAs share common chromatin patterns with protein coding genes in HMEC cell line.

The genomic regions around the TSS (-2kb to 2kb) were divided into bins with 20bp, and the number of reads in each bin was counted. Then the average read density of all genes and lncRNAs were computed for each bin. And we computed the Pearson correlation coefficient of the gene and lncRNA using the averge read density across 200 bins in figure 4A.

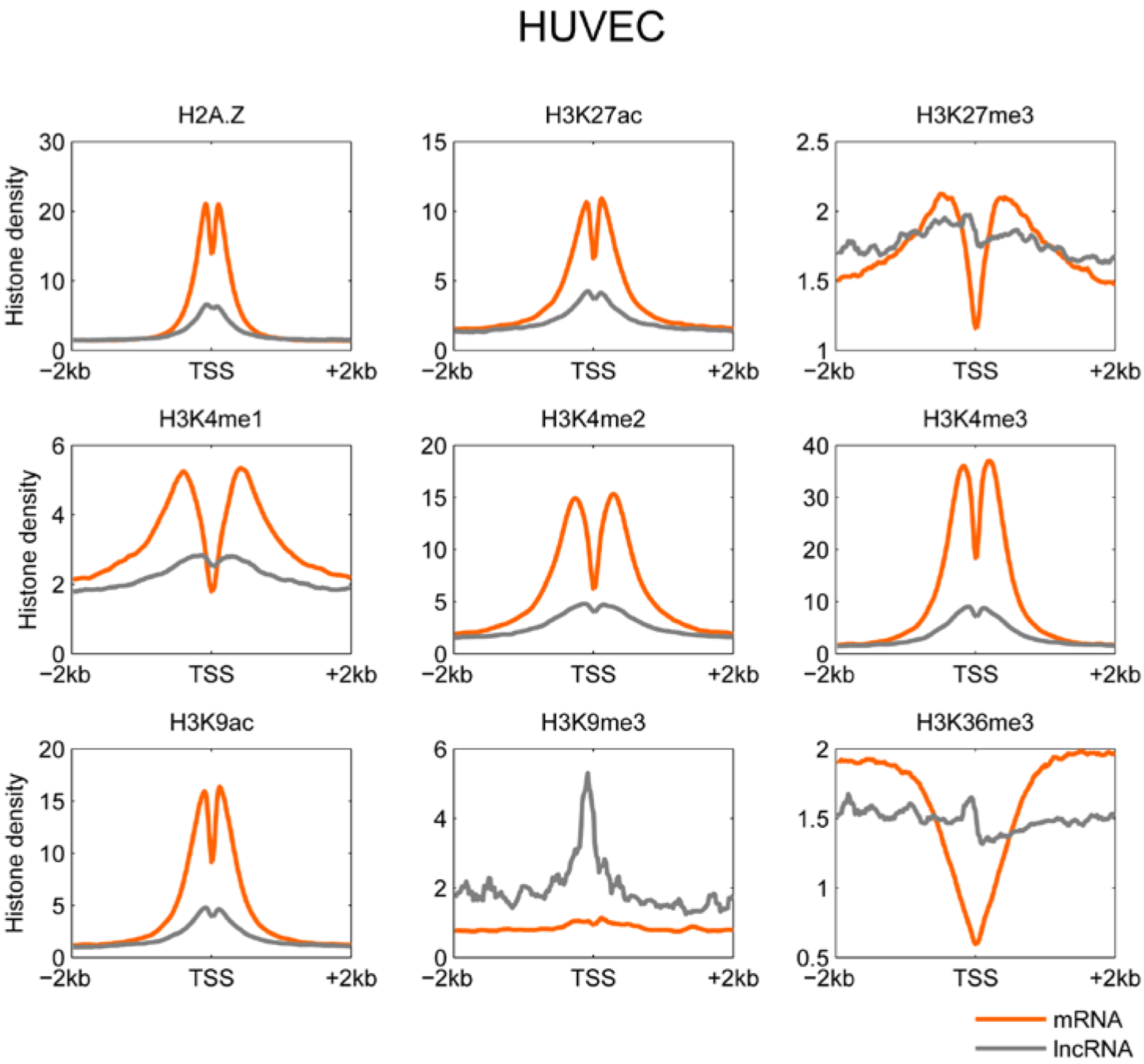

Figure S7 - The lncRNAs share common chromatin patterns with protein coding genes in HUVEC cell line.

The genomic regions around the TSS (-2kb to 2kb) were divided into bins with 20bp, and the number of reads in each bin was counted. Then the average read density of all genes and lncRNAs were computed for each bin. And we computed the Pearson correlation coefficient of the gene and lncRNA using the averge read density across 200 bins in figure 4A.

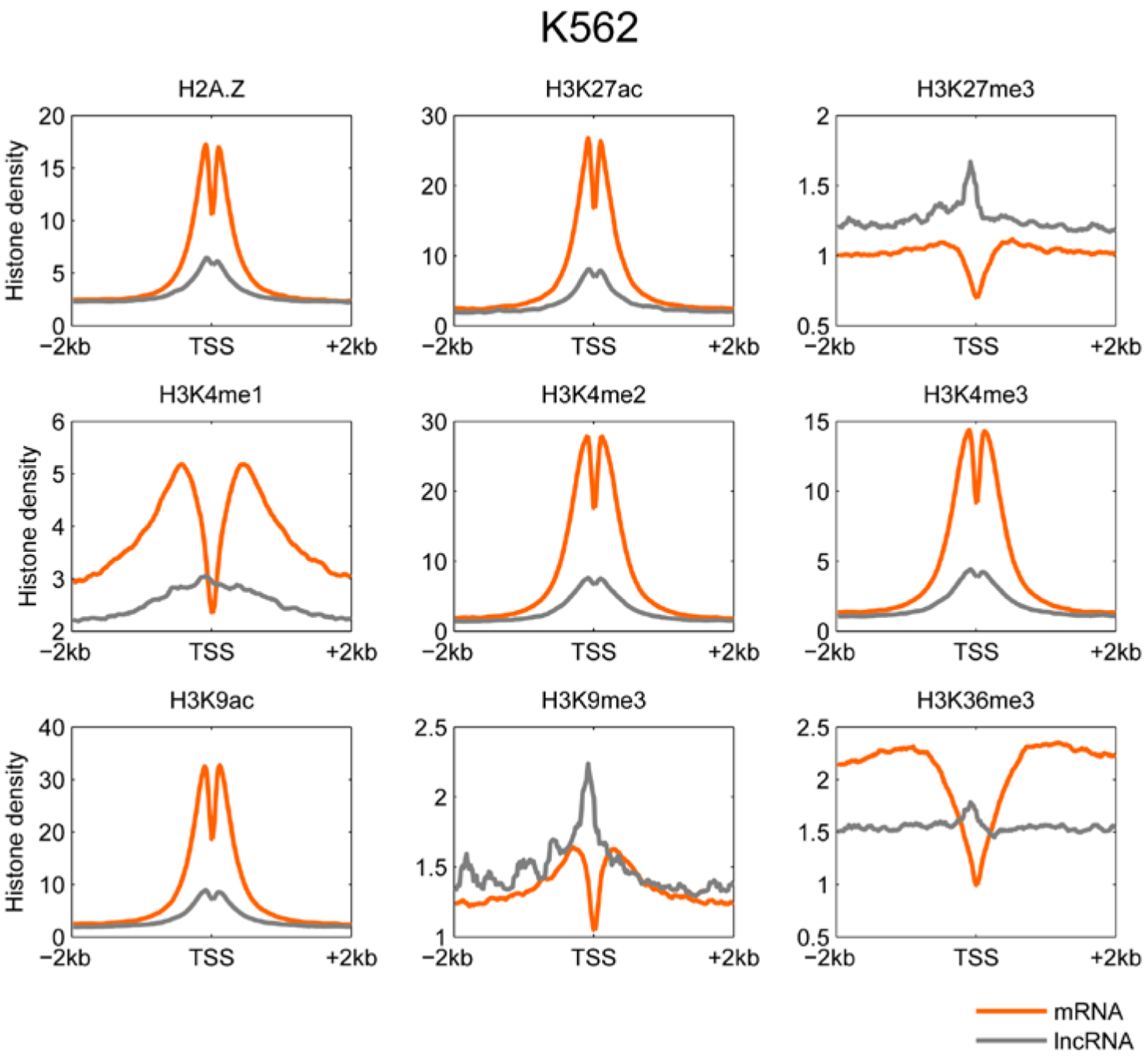

Figure S8 - The lncRNAs share common chromatin patterns with protein coding genes in K562 cell line.

The genomic regions around the TSS (-2kb to 2kb) were divided into bins with 20bp, and the number of reads in each bin was counted. Then the average read density of all genes and lncRNAs were computed for each bin. And we computed the Pearson correlation coefficient of the gene and lncRNA using the averge read density across 200 bins in figure 4A.

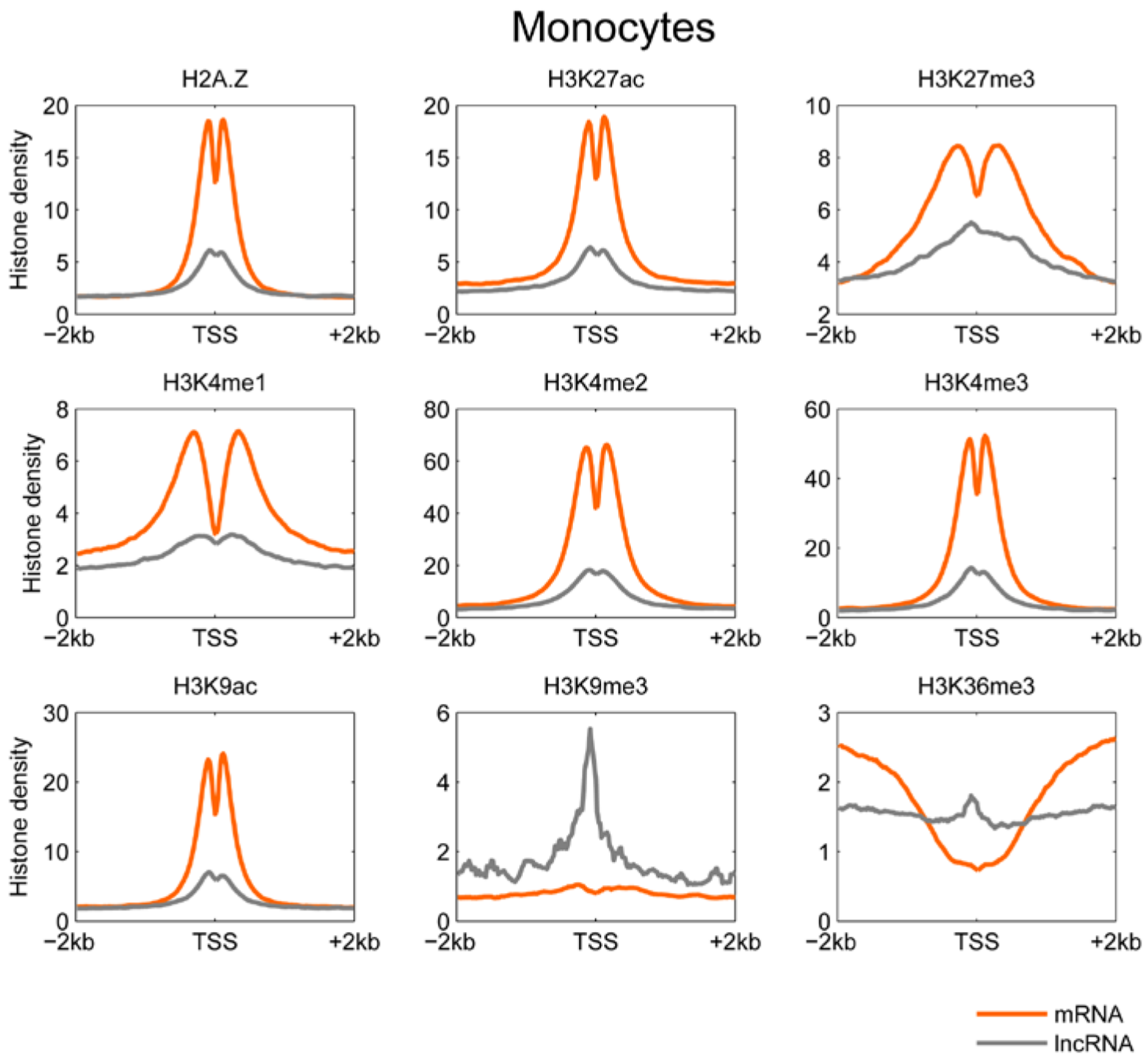

Figure S9 -The lncRNAs share common chromatin patterns with protein coding genes in Monocytes cell line.

The genomic regions around the TSS (-2kb to 2kb) were divided into bins with 20bp, and the number of reads in each bin was counted. Then the average read density of all genes and lncRNAs were computed for each bin. And we computed the Pearson correlation coefficient of the gene and lncRNA using the averge read density across 200 bins in figure 4A.

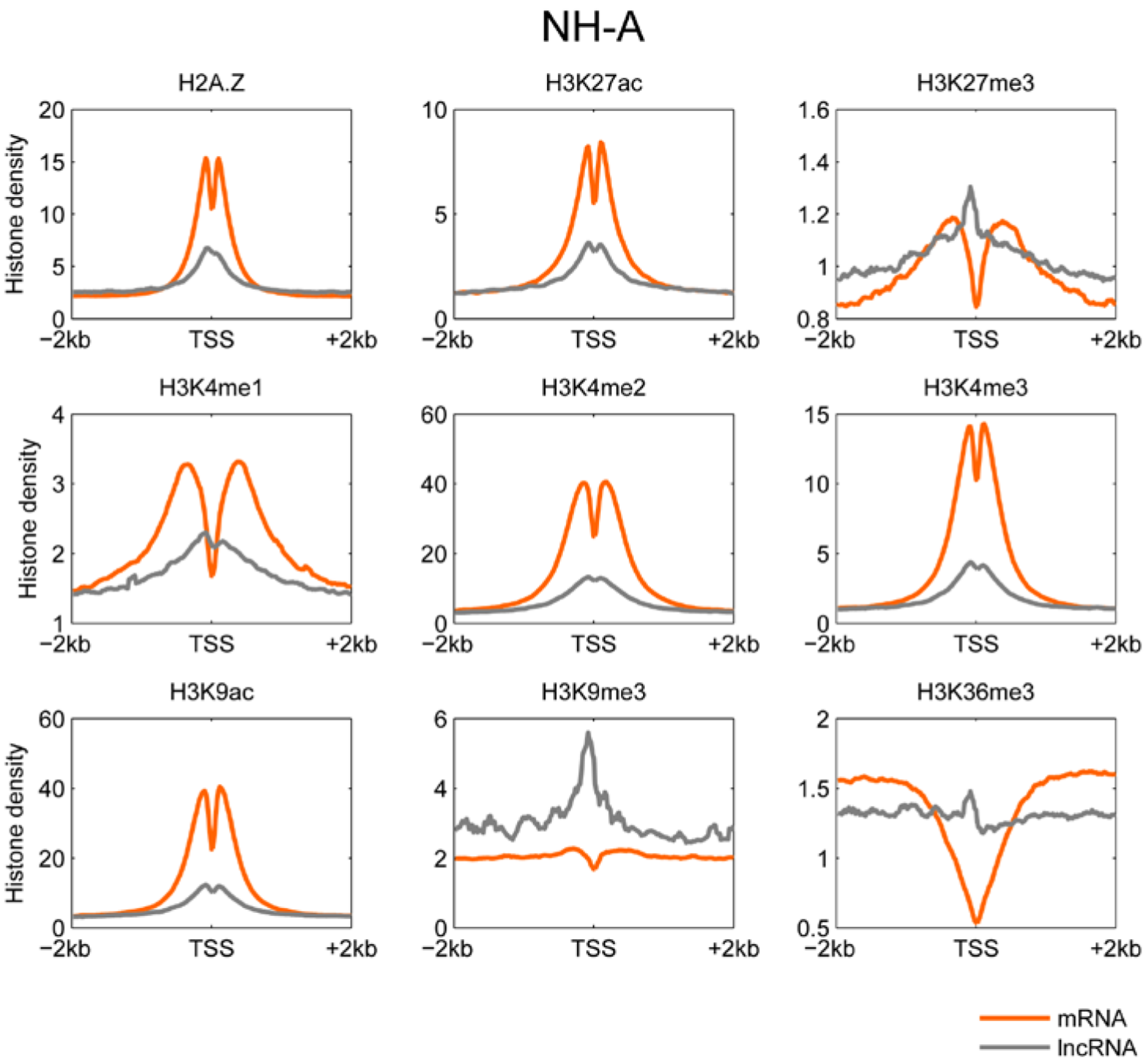

Figure S10 - The lncRNAs share common chromatin patterns with protein coding genes in NH-A cell line.

The genomic regions around the TSS (-2kb to 2kb) were divided into bins with 20bp, and the number of reads in each bin was counted. Then the average read density of all genes and lncRNAs were computed for each bin. And we computed the Pearson correlation coefficient of the gene and lncRNA using the averge read density across 200 bins in figure 4A.

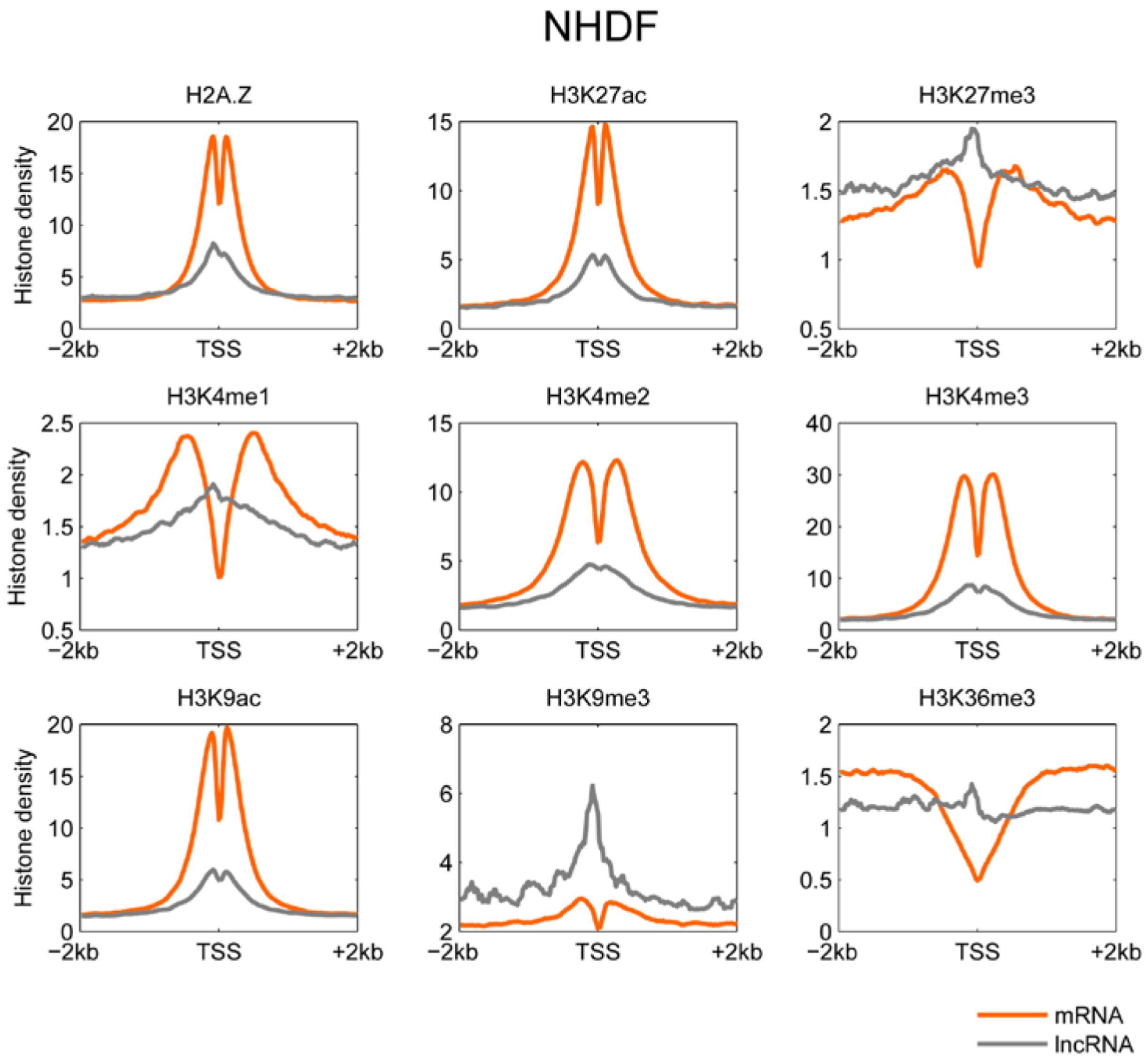

Figure S11 -The lncRNAs share common chromatin patterns with protein coding genes in NHDF cell line.

The genomic regions around the TSS (-2kb to 2kb) were divided into bins with 20bp, and the number of reads in each bin was counted. Then the average read density of all genes and lncRNAs were computed for each bin. And we computed the Pearson correlation coefficient of the gene and lncRNA using the averge read density across 200 bins in figure 4A.

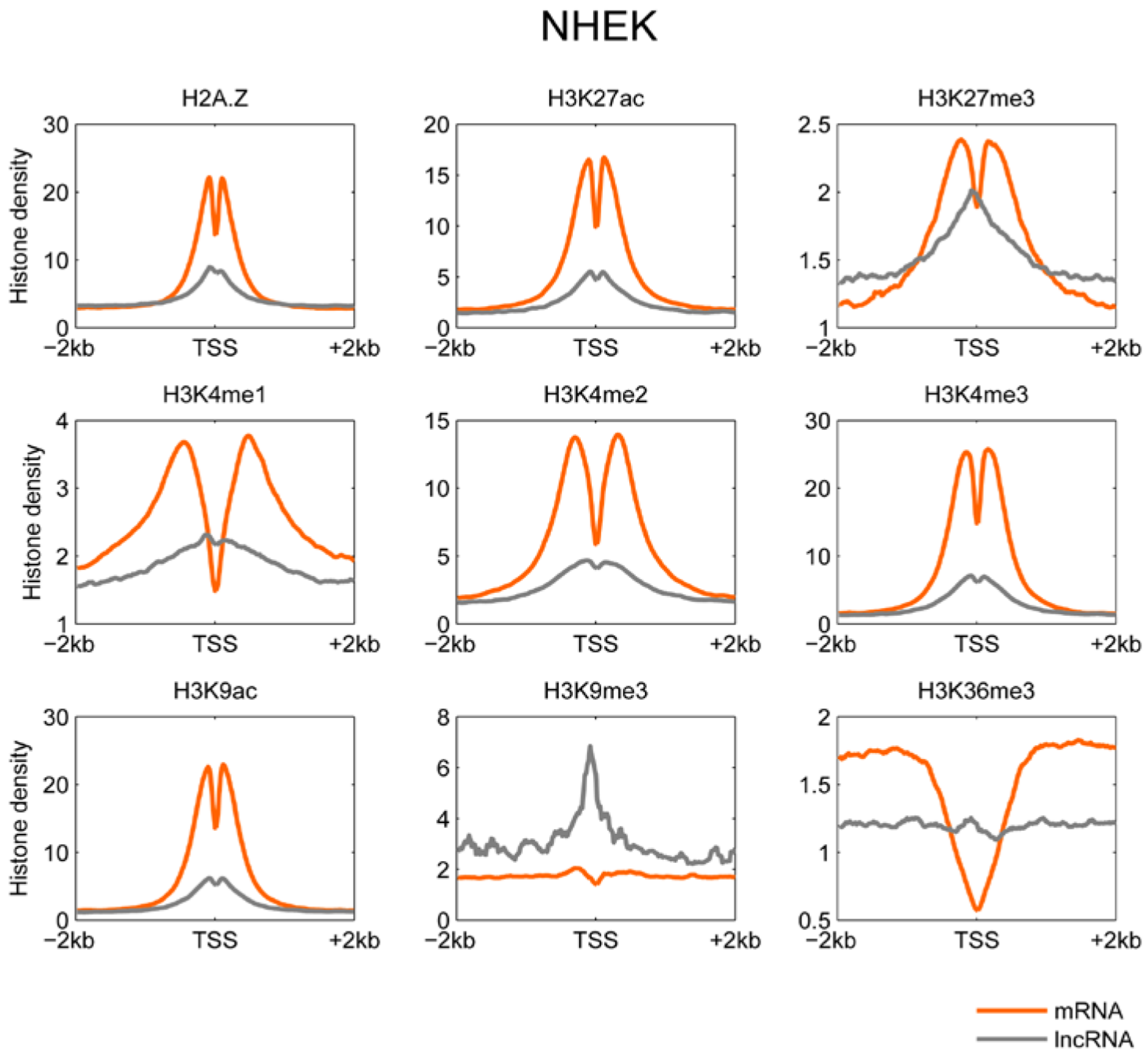

Figure S12 -The lncRNAs share common chromatin patterns with protein coding genes in NHEK cell line.

The genomic regions around the TSS (-2kb to 2kb) were divided into bins with 20bp, and the number of reads in each bin was counted. Then the average read density of all genes and lncRNAs were computed for each bin. And we computed the Pearson correlation coefficient of the gene and lncRNA using the averge read density across 200 bins in figure 4A.

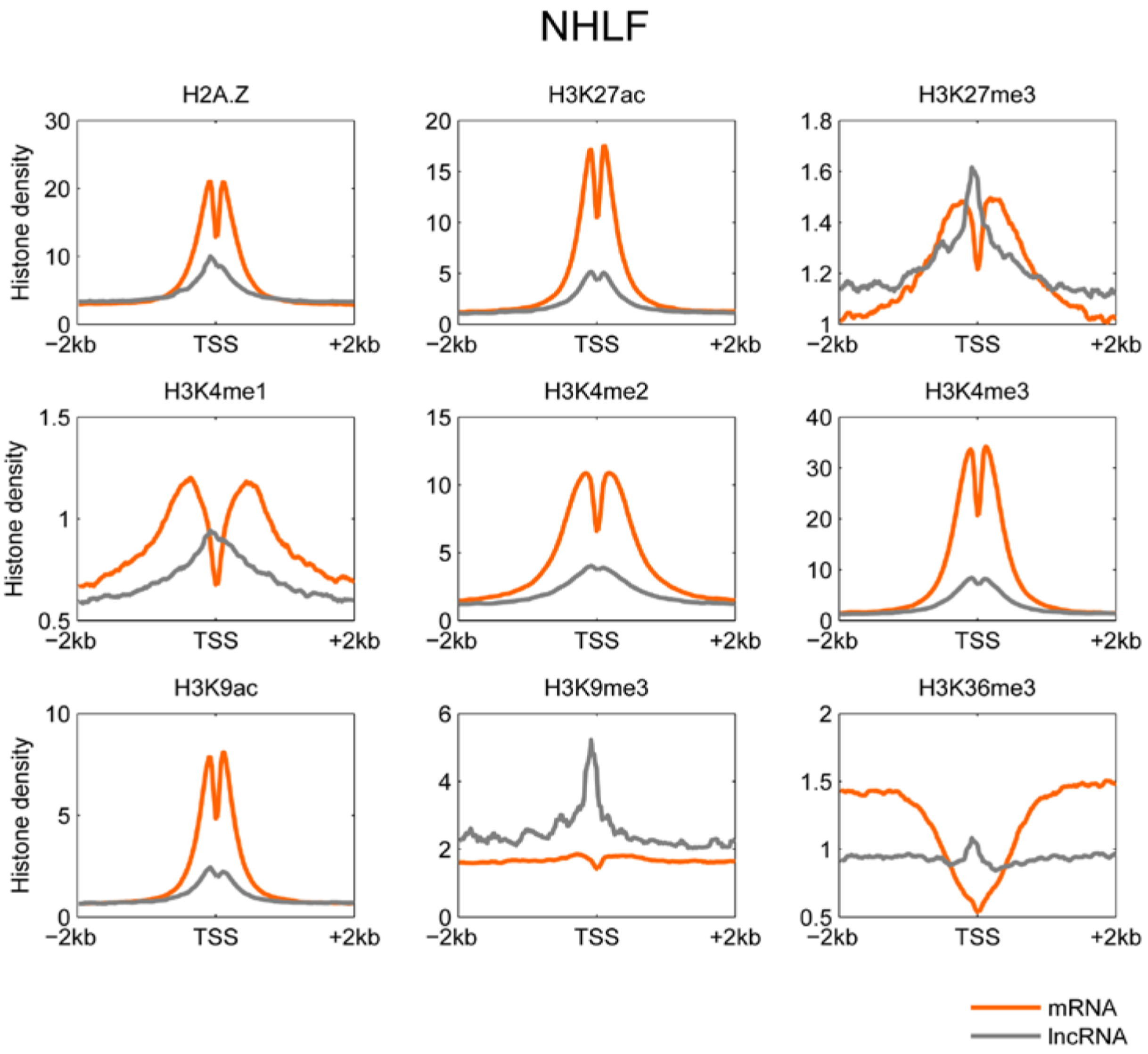

Figure S13 -The lncRNAs share common chromatin patterns with protein coding genes in NHLF cell line.

The genomic regions around the TSS (-2kb to 2kb) were divided into bins with 20bp, and the number of reads in each bin was counted. Then the average read density of all genes and lncRNAs were computed for each bin. And we computed the Pearson correlation coefficient of the gene and lncRNA using the averge read density across 200 bins in figure 4A.

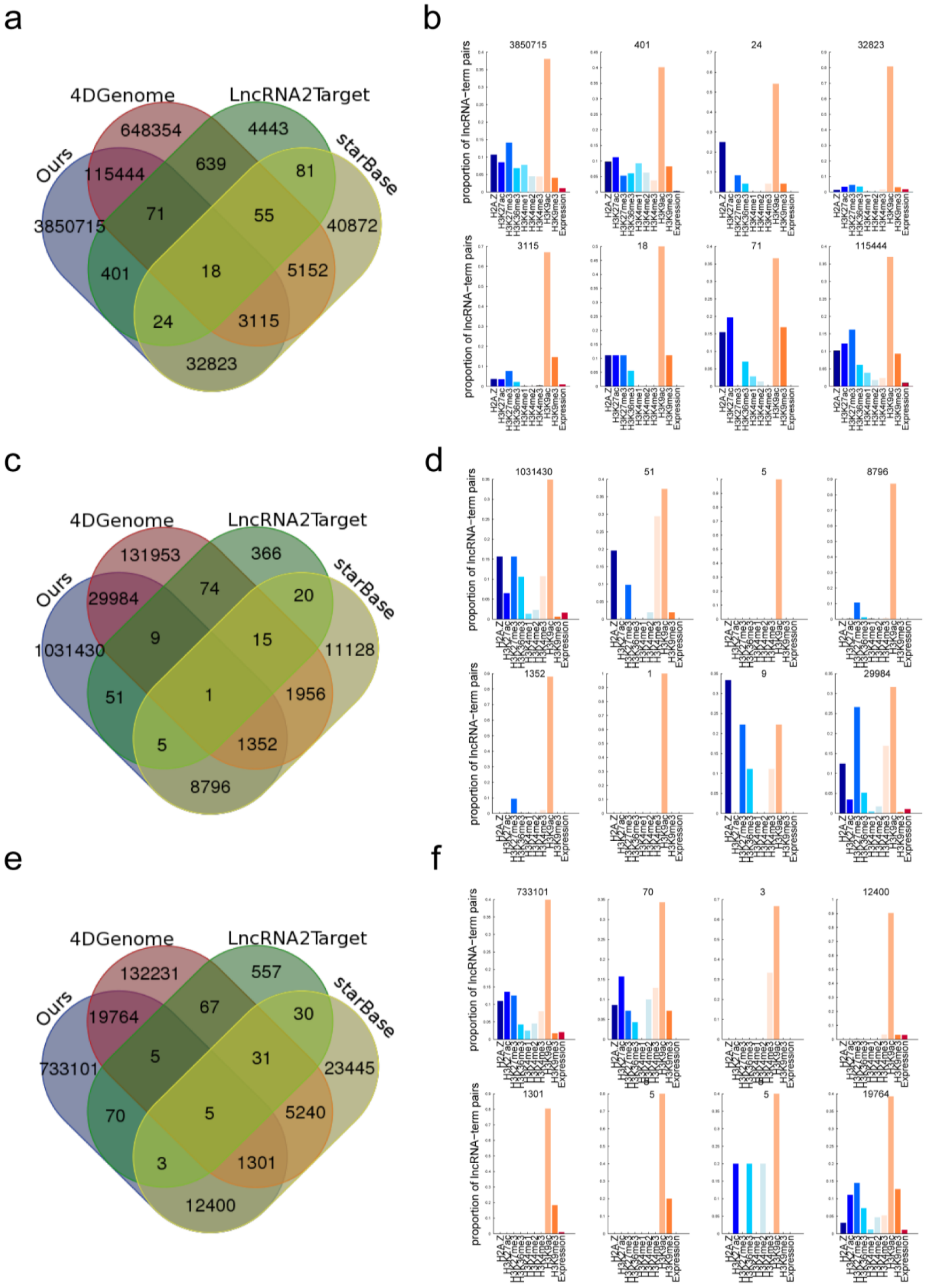

Figure S14-The venn figures show overlap and difference among four methods in terms of lncRNA function prediction.

The Venn diagrams show the overlap and difference between all four methods in terms of lncRNA function prediction in BP, CC and MF respectively (a, c and e). b, d and f show the number of lncRNA-GO term associations predicted by different chromatin and expression features in each subset of the corresponding left Venn diagram.

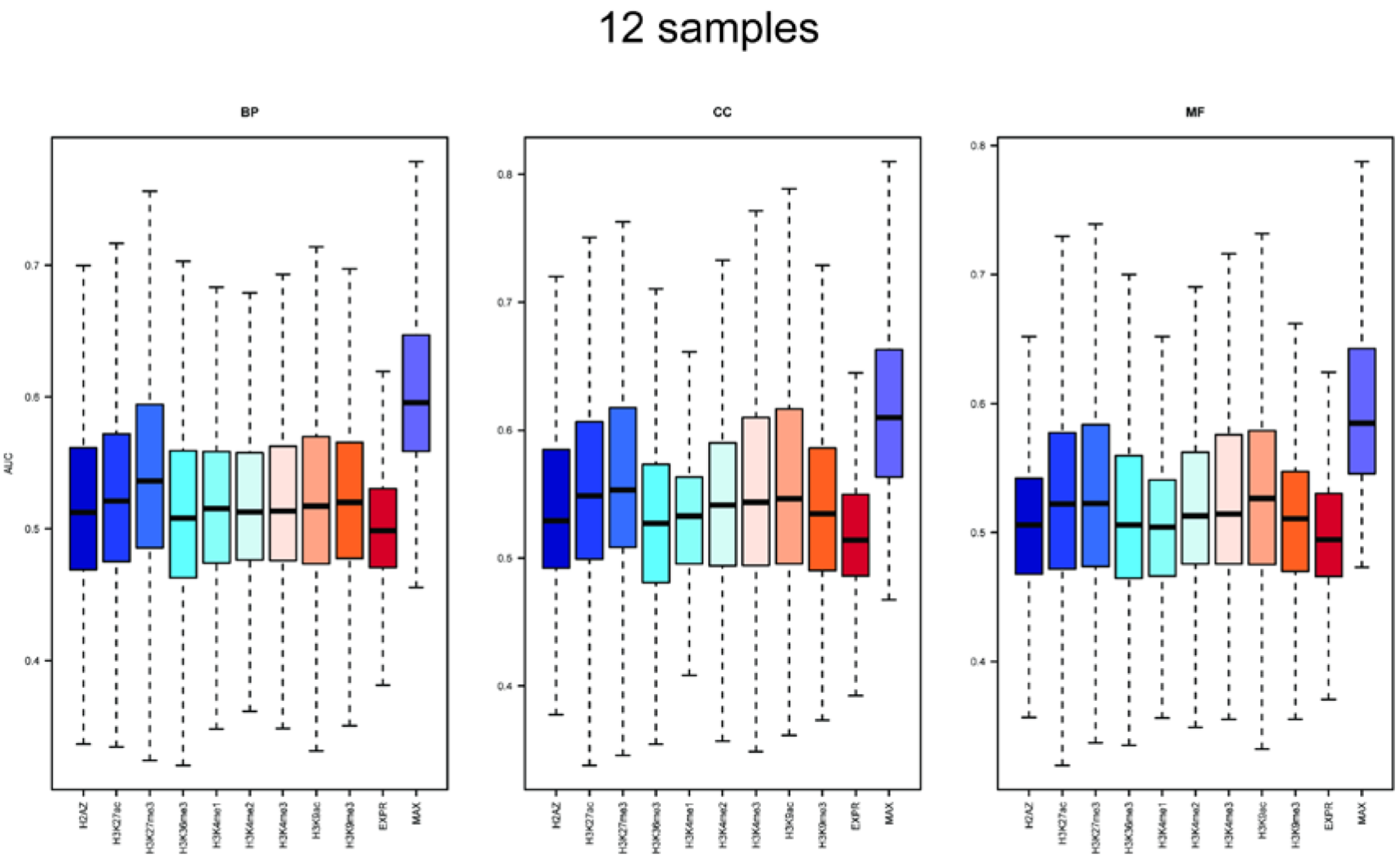

Figure S15 -The prediction power of the models using the signals from different chromatin or expression features using 12 samples.

For each GO term, 100 GSNs with the same number of genes as GSPs were randomly selected from the remaining genes. And then the cross-validation was used to compute the AUC. The average AUCs for 100 times of all GO terms were shown in boxplot. Left panel is for biological process categories, middle panel for cell component categories and the right panel for molecular function categories.

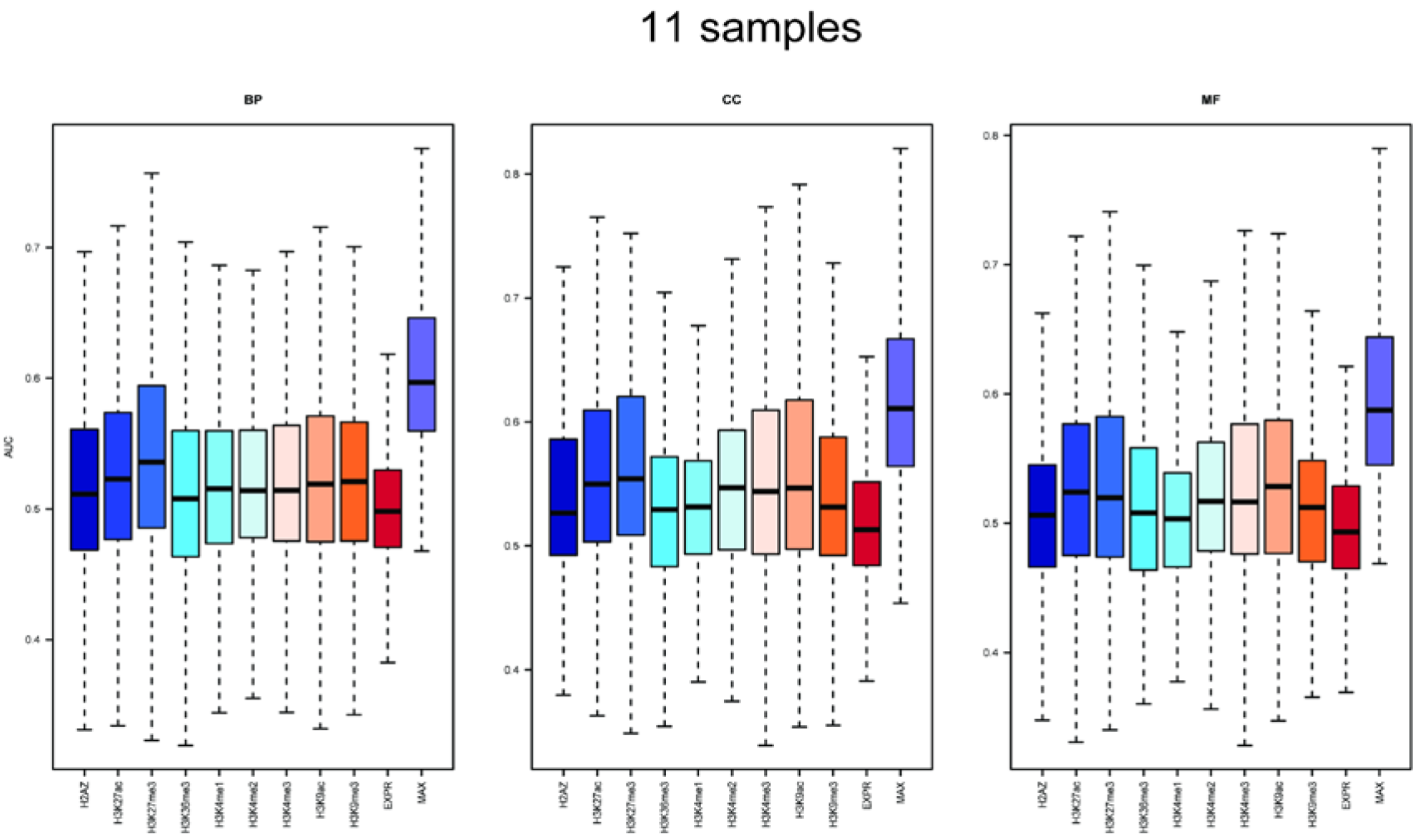

Figure S16 -The prediction power of the models using the signals from different chromatin or expression features using 11 samples.

For each GO term, 100 GSNs with the same number of genes as GSPs were randomly selected from the remaining genes. And then the cross-validation was used to compute the AUC. The average AUCs for 100 times of all GO terms were shown in boxplot. Left panel is for biological process categories, middle panel for cell component categories and the right panel for molecular function categories.

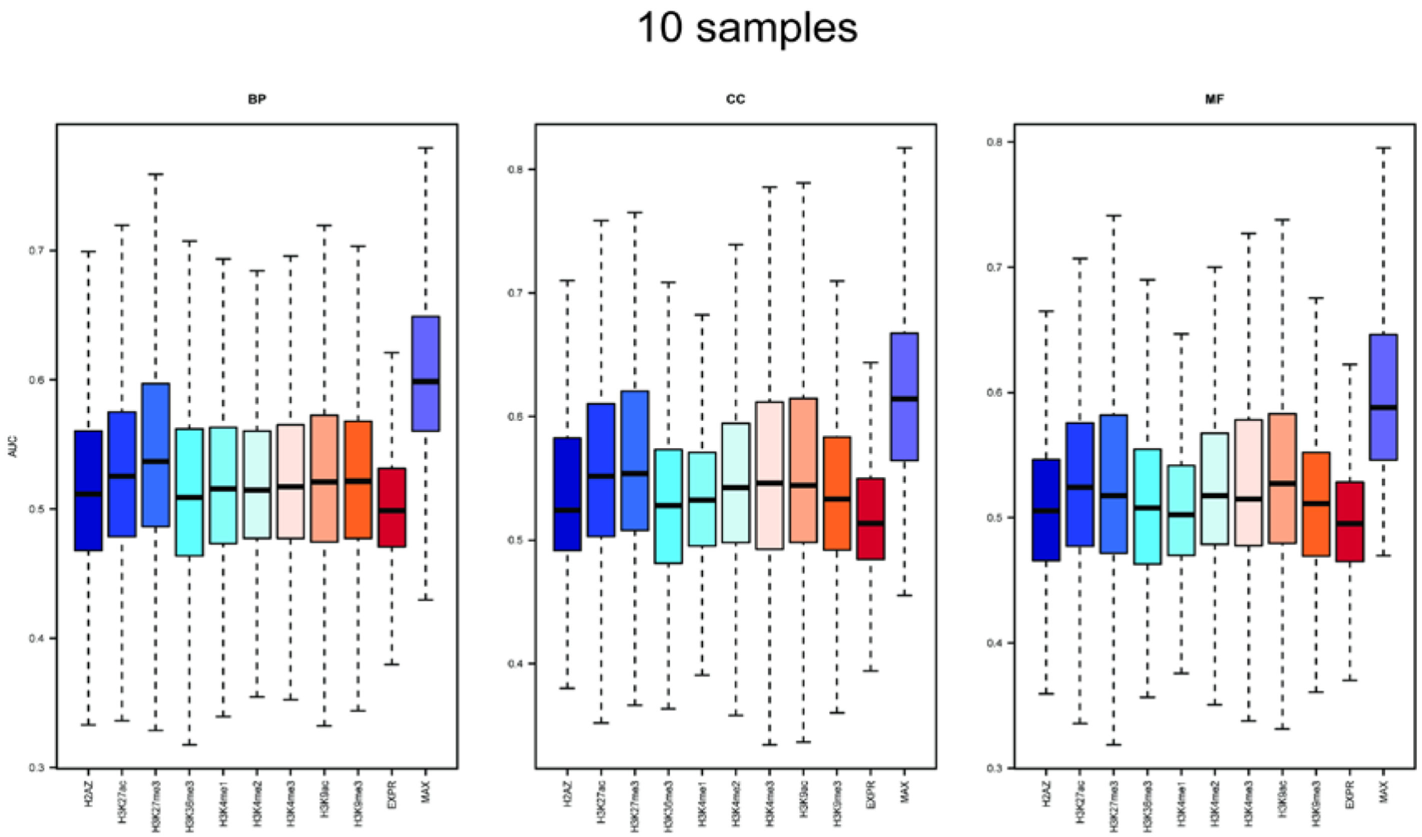

Figure S17 -The prediction power of the models using the signals from different chromatin or expression features using 10 samples.

For each GO term, 100 GSNs with the same number of genes as GSPs were randomly selected from the remaining genes. And then the cross-validation was used to compute the AUC. The average AUCs for 100 times of all GO terms were shown in boxplot. Left panel is for biological process categories, middle panel for cell component categories and the right panel for molecular function categories.

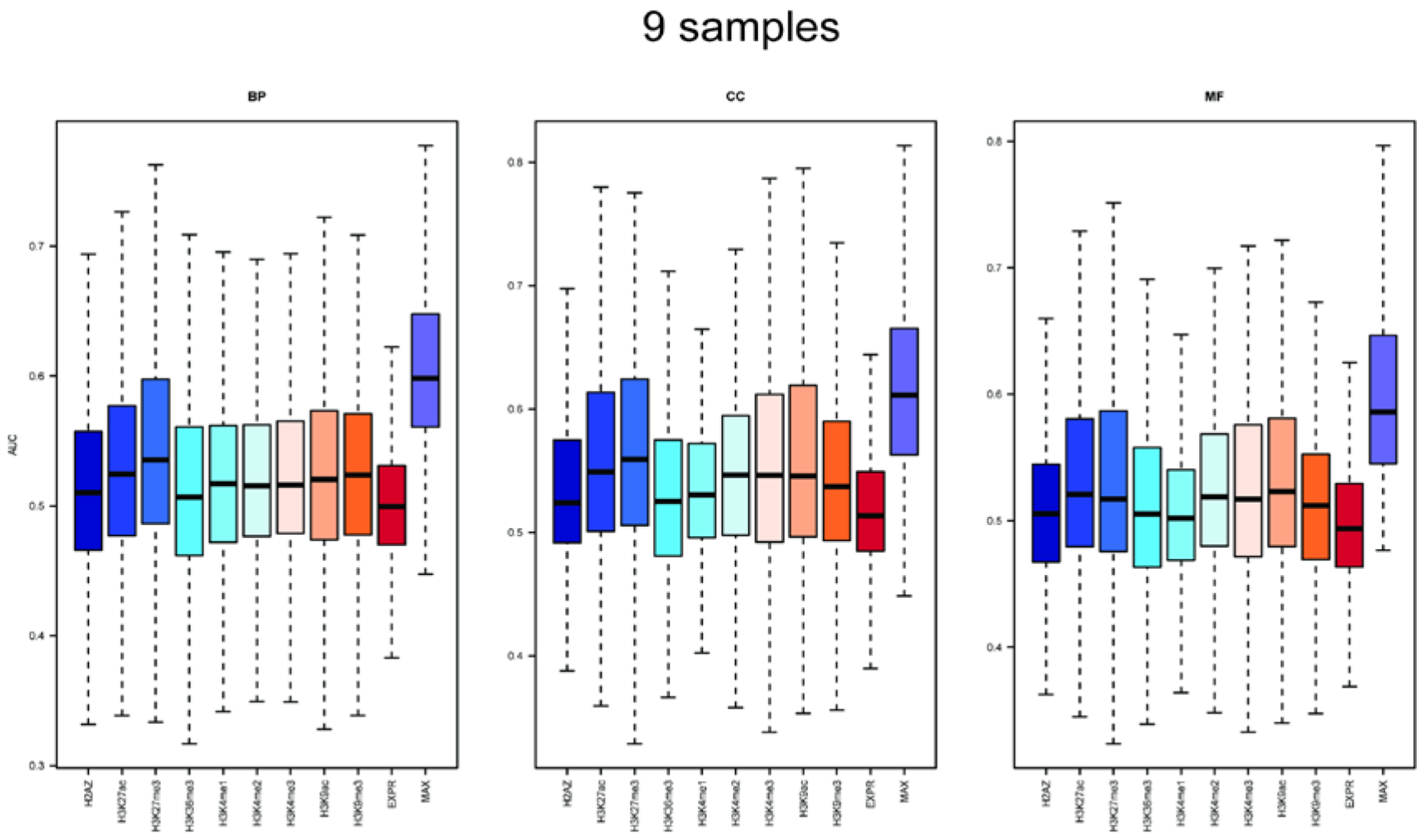

Figure S18 -The prediction power of the models using the signals from different chromatin or expression features using 9 samples.

For each GO term, 100 GSNs with the same number of genes as GSPs were randomly selected from the remaining genes. And then the cross-validation was used to compute the AUC. The average AUCs for 100 times of all GO terms were shown in boxplot. Left panel is for biological process categories, middle panel for cell component categories and the right panel for molecular function categories.
